# High-Throughput ^19^F NMR Chiral Analysis for Screening and Directed Evolution of Imine Reductases

**DOI:** 10.1021/acscentsci.5c00498

**Published:** 2025-06-03

**Authors:** Shucheng Song, Chenyang Wang, Wenjing Bao, Zhenchuang Xu, Jian Wu, Liang Lin, Yanchuan Zhao

**Affiliations:** † State Key Laboratory of Chemical Biology, Shanghai Institute of Organic Chemistry, 12381 University of Chinese Academy of Sciences, 345 Ling-Ling Road, Shanghai 200032, China; ‡ State Key Laboratory of Fluorine and Nitrogen Chemistry and Advanced Materials and Shanghai Hongkong Joint Laboratory in Chemical Synthesis, Shanghai Institute of Organic Chemistry, University of Chinese Academy of Sciences, Chinese Academy of Sciences, 345 Ling-Ling Road, Shanghai 200032, China; § Instrumental Analysis Center, 58309 Shanghai Institute of Organic Chemistry, Chinese Academy of Sciences, Shanghai 200032, China

## Abstract

Biocatalysis is an essential tool for asymmetric synthesis, significantly enhancing the production of chiral molecules. As advancements in protein screening and engineering rapidly evolve, the demand for efficient, rapid chiral analysis methods has intensified. Addressing this need, we introduce a high-throughput ^19^F NMR-based assay that provides comprehensive insights into the enantioselectivity, stereopreference, and yields of biocatalytic reactions. This assay has been successfully applied to screen imine reductases, showcasing its efficacy in the directed evolution for synthesizing an intermediate of the anti-Parkinson drug, rotigotine. Our method offers substantial promise for propelling forward the fields of biocatalysis and synthetic biology by accelerating the assessment of stereochemical outcomes in biocatalytic processes.

## Graphical abstract

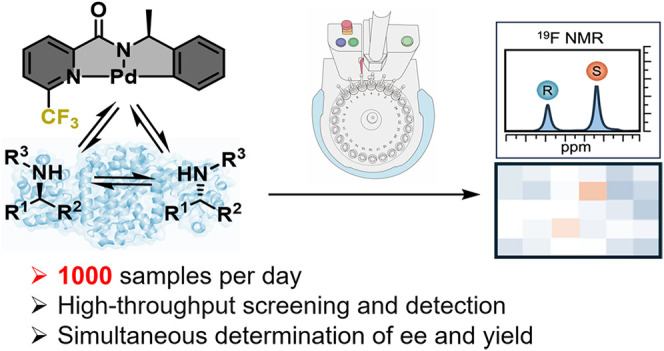

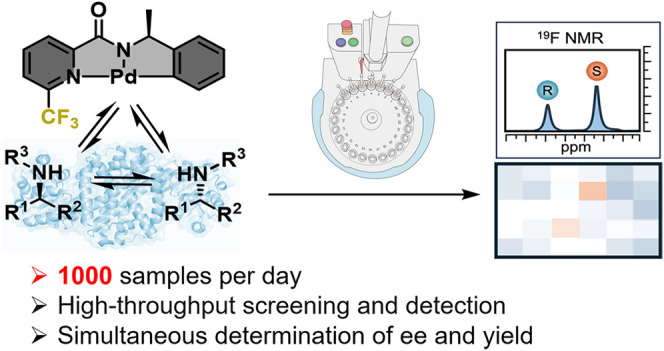

## Introduction

Biocatalysis has emerged as an essential technique across diverse fields, including organic synthesis and pharmaceutical development, valued for its mild reaction conditions, exceptional selectivity—encompassing enantio-, chemo-, and regioselectivity—and its environmental advantages.^1–6^ This approach is particularly effective in asymmetric synthesis of carbon–carbon and carbon-heteroatom bonds. For instance, terpene cyclases efficiently transform unsaturated terpene backbones into cyclic terpenoids with impressive stereoselectivity.^7^ Diels–Alderases facilitate the asymmetric [4 + 2] cyclizations that form cyclohexane rings prevalent in natural products.^8–11^ Moreover, enzymes like cytochrome P450 and iron-α-ketoglutarate-dependent oxygenases (Fe/αKGs) are gaining recognition for their ability to introduce oxygen into unreactive C–H bonds.^12–17^ The field of biocatalysis is rapidly advancing with the integration of directed evolution, which is further bolstered by the use of machine learning and automated processes in reaction setup and workup. Innovations in high-throughput screening technologies have dramatically accelerated enzyme screening, offering rapid, efficient evaluations and shifting away from the traditionally slow pace of enzyme analysis to enhance screening capacity.^18^ Despite these advances, challenges remain in precise chiral screening, where dependence on traditional chiral high-performance liquid chromatography (HPLC) analysis considerably limits the efficiency of the iterative “design-build-test” loop in asymmetric biocatalysis.^19–25^

To enhance the enantioanalysis of biocatalytic reactions, various strategies have been employed. A prominent approach is the use of isotopically labeled pseudoenantiomers or pseudo-*meso*-compounds, as demonstrated by Reetz and colleagues.^26^ This method measures enantioselectivity by comparing the ratio of nonlabeled to labeled products, which can be conveniently determined using techniques such as nuclear magnetic resonance (NMR), and mass spectrometry (Figure 1A).^26,27^ Alternatively, enantioselectivity can be assessed by comparing reaction rates between enantiopure and racemic substrates, with monitoring through changes in fluorescence or heat generation.^28,29^ These methods are effective for screening various kinetic resolutions and desymmetrization reactions (Figure 1A), although they are generally not suitable for reactions involving prochiral substrates. Recently, Kroutil and colleagues have developed an ingenious approach for screening peroxygenases in C–H oxidations, utilizing the formation of nicotinamide adenine dinucleotide phosphate (NADPH) from the highly enantioselective dehydrogenase-catalyzed oxidation of the secondary alcohol product. This serves as a probe for the enantioselectivity of the C–H oxidation (Figure 1B).^30^ Additional sophisticated methods include the use of circular dichroism (CD), which leverages reversible interactions and covalent derivatization of target chiral analytes to induce significant Cotton effects and characteristic CD signals.^31–34^ Despite significant advancements, there remains a significant need for rapid screening methods for amines, which are indispensable in the synthesis of natural products and in pharmaceutical development. Addressing this, the development of specialized enantioanalysis techniques for imine reductases and reductive aminases is crucial. In this regard, we have introduced a novel ^19^F NMR-based assay for rapid screening of biocatalytic reactions producing chiral amines. With NMR spectrometer equipped with a regular autosampler, this method could assess enantioselectivity, stereopreference, and reactivity approximate 1000 samples per day (Figure 1C). By employing this assay in directed evolution experiments, we enhanced the enantioselectivity of a reductive amination reaction to produce an intermediate of the anti-Parkinson drug from 40% enantiomeric excess (ee) to 99%, illustrating its potential to refine biocatalytic methods efficiently.

**Figure 1:**
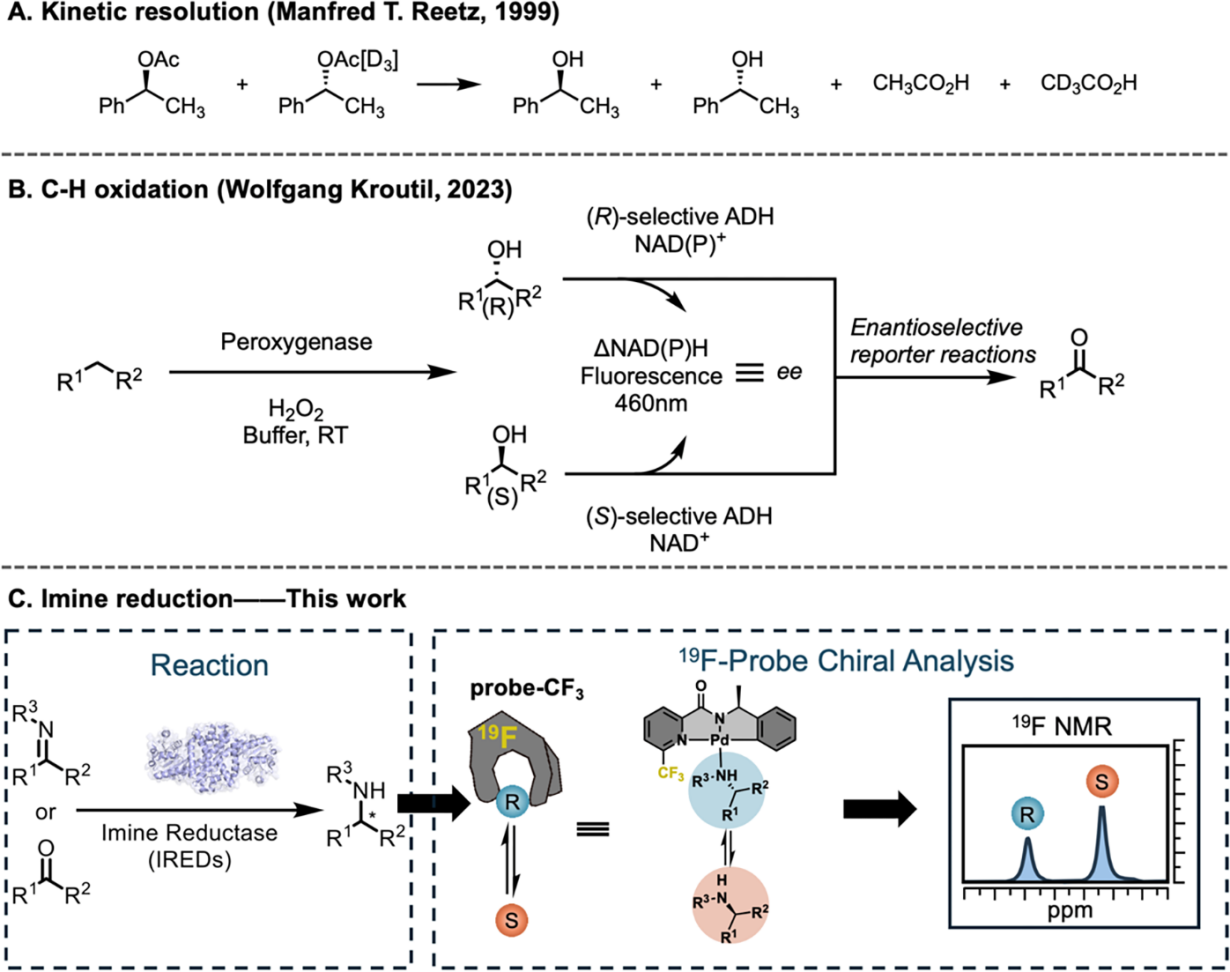
High-throughput chiral detection methods for biocatalysis. (A) Chiral analysis of biocatalytic kinetic resolution enabled by isotope labeling. (B) Fluorescence-based method for chiral analysis of biocatalytic C–H oxidation. (C) ^19^F NMR-based method for chiral analysis of biocatalytic imine reduction.

## Results and Discussion

The rapid enantioanalysis of amines from biocatalytic reactions presents significant challenges. One primary issue is the use of NADPH or its analogs in imine reduction reactions, which absorb ultraviolet (UV) light strongly and can interfere with UV or fluorescence-based detection methods. Such interference often leads to inaccurate correlations between the optical signal and the enantiocomposition. Additionally, the use of cell lysates instead of purified enzymes in biosynthetic screening introduces variability due to their undefined composition, which can cause further interferences. Another major hurdle is the development of rapid chiral analysis methods that are effective for both primary and secondary amines. Secondary amines, in particular, pose a greater challenge due to their increased steric bulkiness,^35–37^ even though they are the desired products in many biocatalytic imine reduction and reductive amination reactions and are prevalent in natural products and critical pharmaceuticals. Moreover, the detection of chiral substances in biocatalytic systems is complicated by low concentrations of chiral products and the presence of substrates, enzymes, and byproducts, which create a complex matrix obscuring detection. This increases the difficulty of accurately detecting and quantifying chiral substances. However, current methods, including fluorescence and CD, often rely on changes in optical signal intensity and do not provide discrete signals for each enantiomer, undermining detection fidelity in complex samples and requiring relatively controlled conditions. To address these challenges, we propose the use of a suitable ^19^F-labeled probe in conjunction with ^19^F NMR, which could provide a viable solution for rapid chiral screening in biocatalytic reactions. A probe that reversibly binds chiral analytes eliminates the need for covalent derivatization, allowing immediate detection upon mixing with the target sample (Figure 1C).^38–40^ The distinct advantages of ^19^F NMR, including high sensitivity and low background noise, provide robust anti-interference capabilities, facilitating rapid detection and quantification of minimal analyte quantities.^41–43^

### Establishment of the Enantioanalysis Platform

To evaluate the viability of our approach, we employed 5-methyl-3,4-dihydro-2*H*-pyrrole (**1**) as a model substrate (Figure 2). Upon reduction, it yields 2-methylpyrrolidine (**1a**) featuring a chiral carbon center connected to the nitrogen atom. Traditional chiral detection of 2-methylpyrrolidine via chromatography is hindered by its weak UV absorption, typically necessitating covalent derivatization followed by chromatographic separation. This process significantly impedes the efficiency of biocatalytic reaction screenings. For the enantioanalysis of 2-methylpyrrolidine, we utilized a chiral ^19^F-labeled cyclopalladium probe (Figure 2A, **probe-CF**_**3**_) characterized by its open binding site, which is essential for accommodating sterically bulky analytes and enhancing the recognition of complex secondary amines.^44^ During detection, the probe interacts with various amine products and the substrate **1**, producing distinct ^19^F NMR signals for each species (Figures 2A–C and Figures S1,3,6 in SI). The enantiomeric composition is accurately reflected in the integrals of these signals. To address the slight variations in the probe’s binding affinity toward the *R* and *S* enantiomers of **1a**, we introduced a correction coefficient to adjust for biases in ee determination arising from differential complexation. This correction factor is readily calculated from the ratio of the integrals of ^19^F NMR signals obtained from the analysis of racemic samples (Figure S2 in SI).^37^ Our investigation has demonstrated that this method can precisely evaluate the enantiocomposition of the target sample, showing an excellent linear correlation between the measured ee values and the actual enantiocomposition (Figure 2D). Notably, this technique typically exhibits a deviation of less than 2%,^39,44^ offering significantly greater accuracy compared to other separation-free enantioanalysis methods. Moreover, the capability of directly detecting the *R* and *S* enantiomers of the amine product enhances the fidelity of the detection process, facilitating the easy identification of impurities or byproducts. This feature is crucial for screening biocatalytic reactions and is difficult to achieve with alternative methods. Evaluating the conversion of biocatalytic reactions is crucial for assessing enzyme performance. While various methods exist to determine the yield of biocatalytic transformations, the simultaneous determination of both yield and enantioselectivity is particularly valuable, as it provides comprehensive information and prevents the oversight of crucial details that might occur if either efficiency or selectivity is screened in isolation. Accurate yield determination using chiral dynamic ^19^F NMR probes has long been challenging, as incomplete binding between the probe and the analyte can lead to significant errors when relying on direct integration of the ^19^F NMR signals. To address this limitation and facilitate the quantification of conversion, (*R*)-2-methylpiperidine (**Int-I**) was introduced as an internal standard (Figure 2C and Figure S3 in SI). This compound competes with the target product for the binding site of **probe-CF**_**3**_. By comparing the relative integrals of the ^19^F signals of the internal standard to those of the product enantiomers, both conversion and enantioselectivity can be simultaneously determined (Figures 2D, 2E and Figures S1 to S5 in SI). This method enhances the accuracy and comprehensiveness of our enzyme screening process, allowing for a more effective evaluation of biocatalytic reactions.

**Figure 2:**
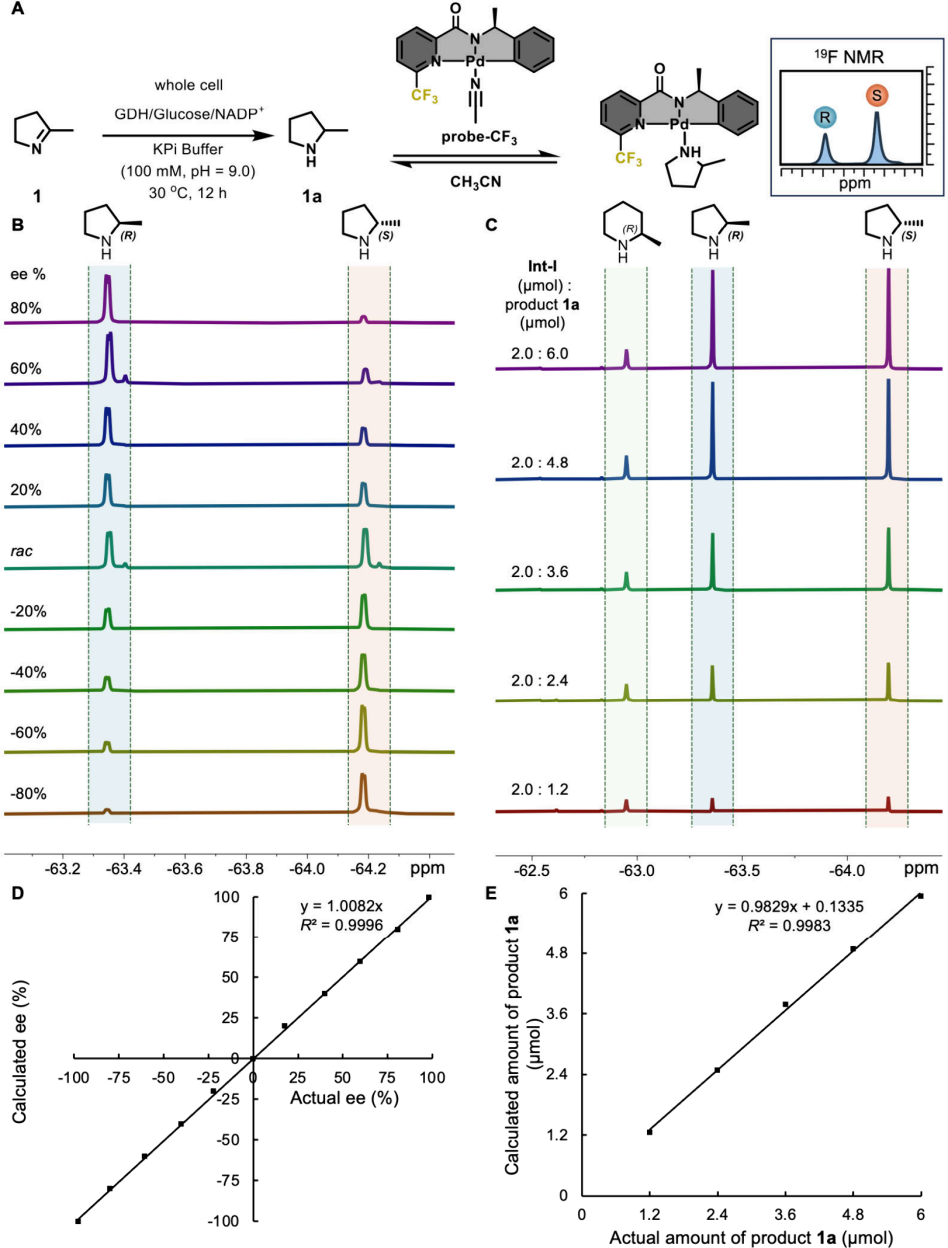
Determination of the ee values and yield using **probe-CF**_**3**_. (A) Schematic representation of the imine reduction reaction and ^19^F NMR-based detection methodology. (B) ^1^H-decoupled ^19^F NMR spectra (16 scans each) of a mixture of **probe-CF**_**3**_ (ca. 9 μmol) in CDCl_3_ and 2-methylpyrrolidine (ca. 6 μmol) of varying enantiocompositions. (C) ^1^H-decoupled ^19^F NMR spectra (16 scans each) of a mixture of **probe-CF**_**3**_ (ca. 9 μmol) in CDCl_3_ and varying amounts of 2-methylpyrrolidine (ranging from 0 to 6 μmol) and 2 μmol of (R)-2-methylpiperidine (**Int-I**) as an internal standard. (D) Calibration plot showing the linear relationship between the measured and actual ee values. (E) Calibration plot depicting the linear correlation between the measured and actual amounts of 2-methylpyrrolidine.

### Screening of Imine Reductases

Having established methods to determine both conversion and enantioselectivity, we next applied this approach to the high-throughput screening of imine reductases for the reduction of specific substrates. Initially, we employed 12 of well-characterized enzymes^45–56^ as query sequences to conduct the basic local alignment search tool (BLAST) search against the nonredundant protein database hosted by the national center for biotechnology information (NCBI). Based on sequence similarity, we compiled a panel of 134 imine reductases (IREDs) (Table S11 in SI). These IREDs were then systematically tested to identify those that exhibited the desired catalytic activity and enantioselectivity. Following the enzymatic reactions (carried out in 96-well plates), the reaction mixture was combined with a deuterated chloroform solution containing **probe-CF**_**3**_ and an internal standard (**Int-I**). After thorough mixing and centrifugation, the deuterated chloroform phase was directly subjected to ^19^F NMR analysis without further purification (Figure 3A). This process allows for the simultaneous evaluation of both yield and enantioselectivity based on the newly generated ^19^F NMR signals (Figure 2). Due to the high sensitivity of this method, only 6 μmol of substrate is required for the analysis, aligning well with standard biocatalytic screening setups. The platform is highly efficient, taking less than 1.5 min to determine both conversion and enantioselectivity for each reaction (Table S6 in SI). With an NMR spectrometer equipped with a standard autosampler, 24 samples can be analyzed in 35 min, requiring no further optimization of the spectroscopy or acceleration of the autosampling process. This setup allows for the screening of approximately 1,000 samples in a single day. Chromatographic resolution of chiral analytes typically takes 10 to 40 min per sample and often necessitates covalent derivatization for amine analysis (Figure S20). Our approach boosts analytical efficiency by at least 10 to 20 times, offering substantial potential to advance biosynthetic techniques. During the screening process, our platform swiftly identified that out of 134 IREDs, 79 demonstrated activity in reducing the substrate **1** to **1a**, exhibiting moderate to high activity levels (Figure 3C and Table S1 in SI). These enzymes showed considerable stereoselectivity, with the majority favoring the production of (*R*)-2-methylpyrrolidine. Notably, only three enzymes—IRED-186, IRED-191, and IRED-192—exhibited S-stereoselectivity (Figure 3B and Table S1 in SI). Among these, IRED-191 was particularly notable for its high S-selectivity, achieving an ee of 90% (S) (Table S1 in SI). To elucidate the molecular basis of stereoselectivity in imine reduction, we conducted a multiple sequence alignment of IREDs with R-selectivity and those with S-selectivity obtained through screening (Figure S18 in SI), and performed homology modeling on IRED-191. We then compared the simulated structure of IRED-191 with the known structures of *R*-selective IREDs (Figure S19 in SI).^47,51,55^ Our analysis revealed significant divergence at the catalytic residue. Aspartic acid (Asp) is consistently conserved in the active sites of *R*-selective IREDs. In contrast, the equivalent position in *S*-selective enzymes is predominantly occupied by either tyrosine (Tyr) or asparagine (Asn). This variation suggests a pivotal role in controlling enantioselectivity. This correlation between the identity of the catalytic residue and the enzyme’s stereoselectivity underscores the significance of this position in defining both the catalytic mechanism and the stereochemical outcome. We further validated these findings using chiral HPLC. The ee values of 2-methylpyrrolidine **1a** produced by IRED-181, IRED-185, and IRED-188, determined via chiral HPLC (which required derivatization and a 40 min chromatographic separation), were consistent with those measured by our ^19^F NMR platform. The deviation between the two methods was less than 1% (Figure S20–S24 and Table S5 in the SI), demonstrating the accuracy and reliability of our ^19^F NMR approach. Notably, our method is fully compatible with whole-cell-based enzymatic assays, as demonstrated by experiments showing that the ee values of products generated by IRED-191-expressing whole cells and corresponding cell lysates differed by less than 1% (Table S7 in SI). This analytical platform is not limited to analyzing 2-methylpyrrolidine **1a**; it can easily be adapted for the detection of other amines and N-heterocycles (Figures S12 – S17 and Table S4 in SI). For instance, when screening 1-methyl-3,4-dihydroisoquinoline, we identified 109 IREDs capable of facilitating its reductive transformation, which suggests a broader enzyme applicability for this substrate compared to substrate **1a**. Most enzymes still preferred *R*-selectivity, but nine enzymes (IRED-33, IRED-42, IRED-44, IRED-45, IRED-181, IRED-182, IRED-186, IRED-191, and IRED-192) exhibited *S*-selectivity. Remarkably, all *S*-selective enzymes except IRED-186 displayed excellent *S*-selectivity, with ee values exceeding 99% (Table S4 in SI). The findings underscore the versatility and efficiency of our NMR-based platform in rapidly screening a wide array of biocatalysts for both activity and selectivity across different substrates, highlighting its utility in accelerating the development of efficient, selective biocatalysts.

**Figure 3:**
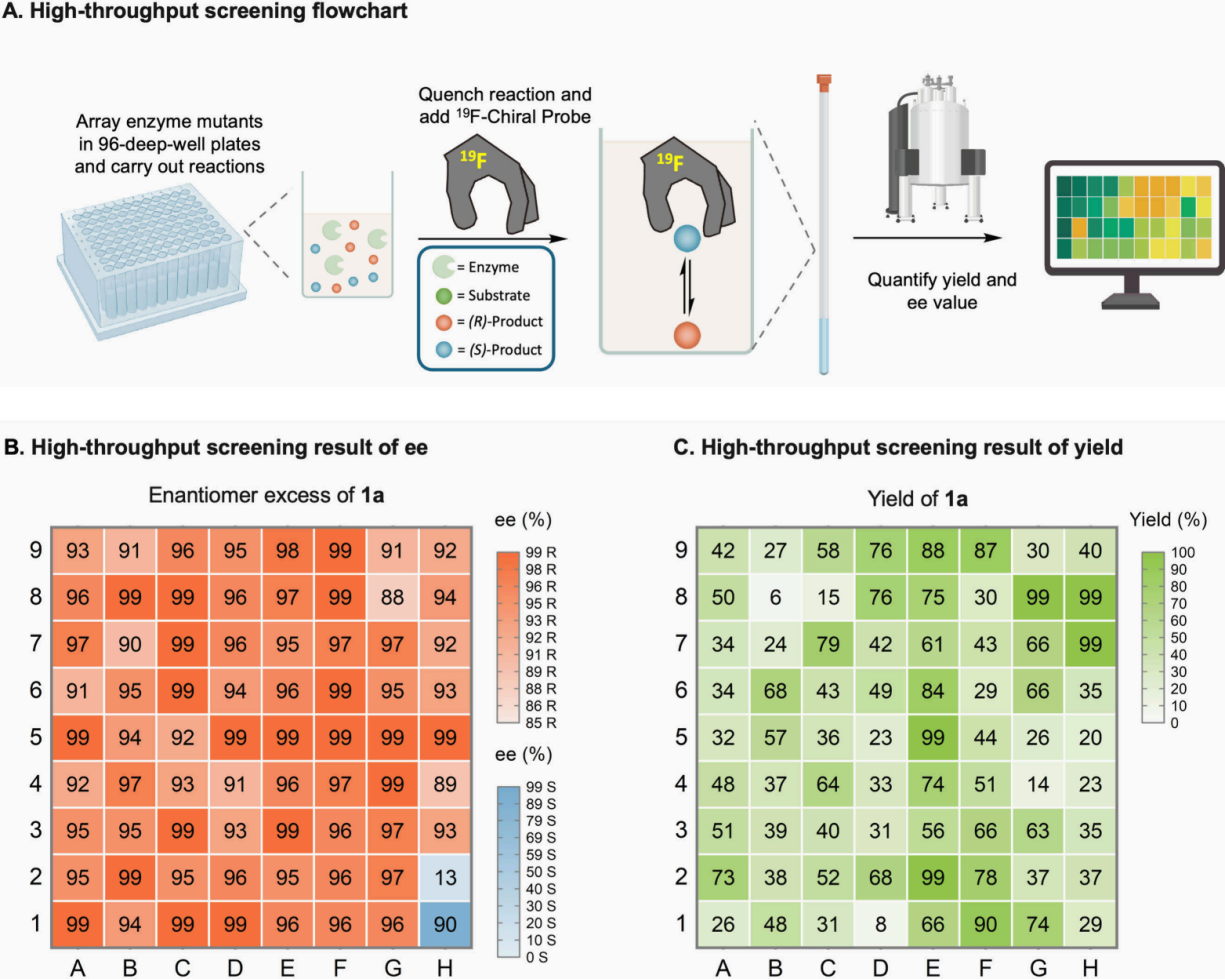
(A) Schematic workflow of the high-throughput NMR chiral screening process. (B) Enantioselectivities of the screened biocatalytic reactions, determined by ^19^F NMR analysis. (C) Yields of the screened biocatalytic reactions, quantified by ^19^F NMR relative to an internal standard. Reaction conditions: 300 μL reaction volume, substrate **1** (20 mM), NADP^+^ (0.5 mM), BmGDH (1 mg/mL), d-glucose (30 mM), KPi buffer (100 mM, pH 9.0), 800 rpm, 30 °C, 12 h. The product yield was quantified by ^19^F NMR analysis relative to an internal standard. The enantiomeric excess (ee) values were determined using ^19^F NMR.

### Directed Evolution of Imine Reductases

After establishing our screening platform and validating its performance across various template substrates, we next sought to demonstrate its broader application by conducting directed evolution targeting high-value compounds, including pharmaceutical intermediates (Figure 4A). Imine reductases are powerful biocatalysts for the asymmetric synthesis of optically pure amines, and recent advances have further underscored their strong potential for industrial application.^48,57–59^ To showcase the utility of our platform, we selected a key precursor to the anti-Parkinson drug rotigotine as a model target. The ability of wild-type enzymes to produce this intermediate with high enantioselectivity was previously demonstrated by Turner and co-workers in 2021.^60^ Given the increasing importance of directed evolution and the necessity to screen large libraries of variants efficiently, this case study serves to validate the effectiveness of our ^19^F NMR-based screening platform and provides a route for accessing highly selective enzymes. The chiral amine component of rotigotine is in the *S*-configuration, prompting us to screen our IRED panel for this specific reaction (Figure 4B). It is noteworthy that propylamine, when used in excess, may compete with product **2a** for the binding site on **probe-CF**_**3**_. Consequently, the residual propylamine was removed under reduced pressure after the reaction to minimize interference. In our investigation, IRED-105, IRED-99, and IRED-195 all exhibited substantial activity; however, only IRED-195 showed the desired S-selectivity. It achieved a high yield coupled with moderate enantioselectivity of 40% ee (Table S2 in SI). These results are consistent with those reported in previous studies.^61^ We then initiated directed evolution on IRED-195 by docking the imine intermediate into the enzyme’s active site (Figure 4C). Docking studies identified nine amino acids within 4 Å of the substrate that potentially interact directly with it: T101, I129, L180, M183, Y184, W214, F240, G248, and F249. We applied saturation mutagenesis to these residues and conducted high-throughput screening using our established NMR platform to assess both activity and stereoselectivity. From screening 171 mutants, the variants G248T and G248Y demonstrated significant improvements in enantioselectivity, increasing from 40% (*S*) in the wild type to 90% (*S*), while maintaining the original activity levels (Figure 4D and Table S3 in SI). Mutating I129 to a more rigid proline notably enhanced S-selectivity. In contrast, substituting it with bulkier amino acids such as phenylalanine, tyrosine, tryptophan, or even smaller cysteine resulted in decreased S-selectivity (Figure 4D). The residue G248 proved critical for enhancing S-selectivity, with mutations to bulkier amino acids consistently leading to significant improvements. The enhancement in selectivity by mutations at G248 was not previously reported, likely because past analytical strategies did not simultaneously screen for conversion and enantioselectivity. To further improve the enantioselectivity of the enzyme, we conducted additional rounds of screening using iterative saturation mutagenesis (ISM).^62,63^ Following iterative optimization, the final mutant, IRED-195-R2, was able to synthesize the target intermediate with 99% ee (S) and 72% yield on a 2.5 mmol scale (Table S9 in the SI). Collectively, our approach enables us to elucidate the relationship between enzyme activity, selectivity, and individual residues with greater precision. The robust enhancement in selectivity underscores the efficacy of our high-throughput screening platform for directed evolution.

**Figure 4:**
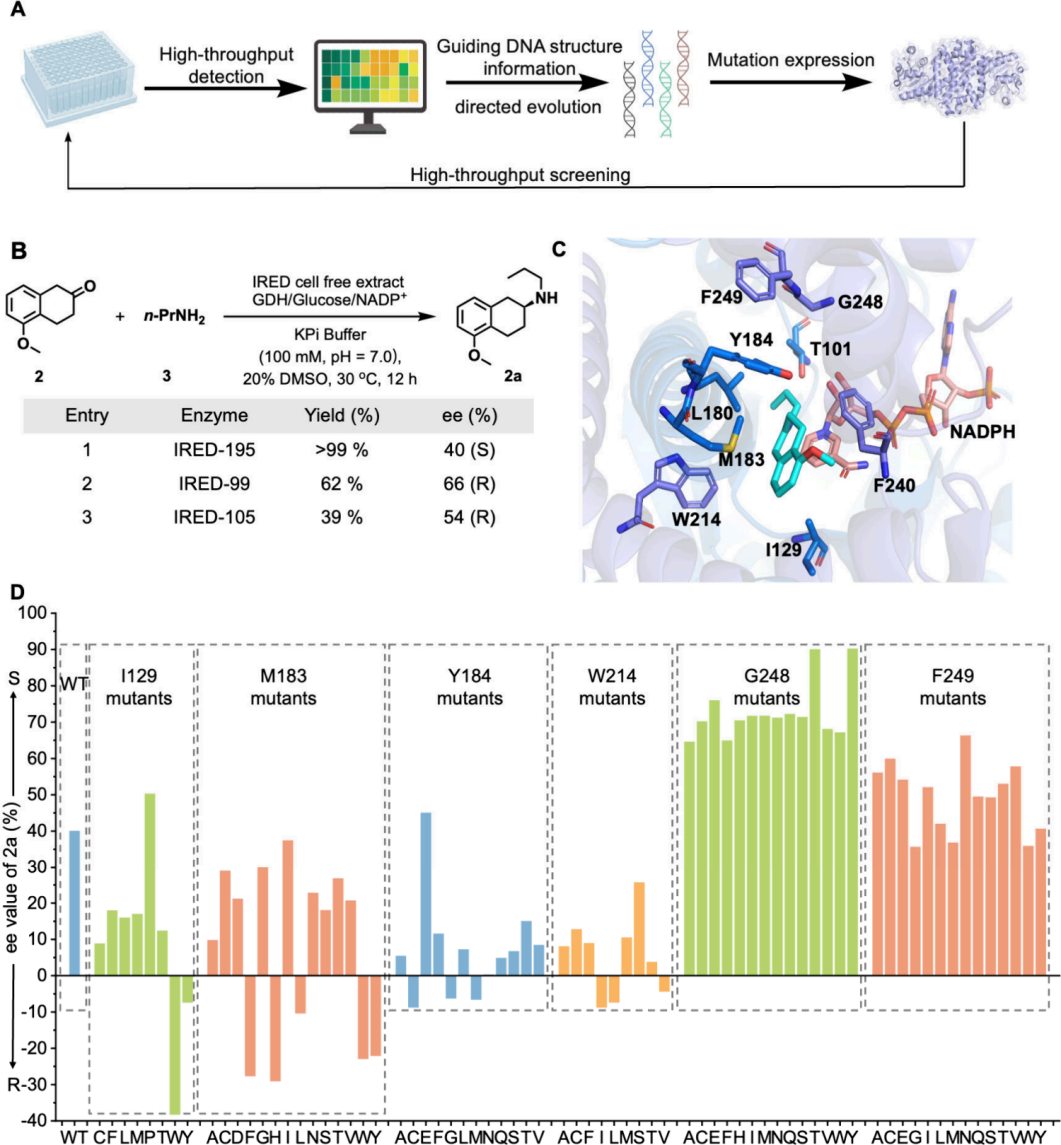
(A) Workflow of the directed evolution process. (B) Reaction scheme for the reductive amination reaction and enzyme screening results. Reaction conditions: 500 μL reaction volume, cell-free extract 200 μL, substrate **2** (10 mM), n-propylamine (1 M), NADP^+^ (0.5 mM), BmGDH (1 mg/mL), d-glucose (30 mM, 3 equiv), KPi buffer (100 mM, pH 7.0), DMSO (20%, v/v), 800 rpm, 30 °C, 12 h. The product yield was quantified by ^19^F NMR analysis relative to an internal standard. The enantiomeric excess (ee) values were determined using ^19^F NMR. (C) Molecular docking of the intermediate of the reductive amination of substrates **2** and **3** into the active cavity of IRED-195. (D) Enantioselectivity of IRED-195 (wild-type, WT) and its mutants at residue positions 129, 183, 184, 214, 248, and 249.

## Conclusions

In summary, we have developed a novel ^19^F NMR-based high-throughput platform for screening imine reductases. This approach outperforms traditional chiral chromatographic analysis, achieving a more than 10-fold improvement in time efficiency for enantioanalysis. It allows for the simultaneous determination of yields and enantioselectivity, providing a more comprehensive data set compared to methods that only measure conversion or enantioselectivity. Capable of screening over 1,000 biosynthetic reactions, this platform is well-suited for use in directed evolution processes. Previous directed evolution workflows typically prioritize initial screening for catalytic activity, with enantioselectivity assessed only in subsequent stages. In contrast, by simultaneously monitoring both conversion and enantioselectivity, our method not only identifies variants with improved activity and selectivity but also captures mutants that exhibit enhanced stereocontrol despite reduced activity. This integrated approach enables a more nuanced and comprehensive strategy for biocatalyst engineering, where even ″suboptimal″ variants provide valuable mechanistic insights that can inform the rational design of enzymes balancing both activity and stereochemical precision. Moreover, its application extends beyond imine reductases to include other enzymatic reactions, such as those involving chiral alcohols, nitriles, and sulfoxides, contingent upon the use of a ^19^F-labeled probe with appropriate recognition properties. The ability to generate distinct ^19^F NMR signals for each enantiomer ensures excellent compatibility with complex matrices. This capability paves the way for the screening of more intricate biological processes and establishes the platform as a robust tool for advancing enzyme engineering and biocatalysis research.

## Supplementary Material



## Notes

General information about materials and instruments, procedures for NMR experiments, NMR spectra, HPLC traces; details of analytical protocols and bioinformatics data (PDF)

## Abbreviations

WT: wild type
IRED: imine reductase
BLAST: Basic Local Alignment Search Tool
NCBI: National Center for Biotechnology information.

## References

[1] Wu S., Snajdrova R., Moore J. C., Baldenius K., Bornscheuer U. T. (2021). Biocatalysis: Enzymatic Synthesis for Industrial Applications. Angew. Chem., Int. Ed..

[2] Bornscheuer U. T., Huisman G. W., Kazlauskas R. J., Lutz S., Moore J. C., Robins K. (2012). Engineering the third wave of biocatalysis. Nature.

[3] Sheldon R. A., Woodley J. M. (2018). Role of Biocatalysis in Sustainable Chemistry. Chem. Rev..

[4] Clouthier C. M., Pelletier J. N. (2012). Expanding the organic toolbox: a
guide to integrating
biocatalysis in synthesis. Chem. Soc. Rev..

[5] Ran N., Zhao L., Chen Z., Tao J. (2008). Recent applications
of biocatalysis in developing green chemistry for chemical synthesis
at the industrial scale. Green Chem..

[6] Winkler C. K., Schrittwieser J. H., Kroutil W. (2021). Power of Biocatalysis for Organic
Synthesis. ACS Cent. Sci..

[7] Christianson D. W. (2017). Structural
and Chemical Biology of Terpenoid Cyclases. Chem. Rev..

[8] Auclair K., Sutherland A., Kennedy J., Witter D. J., Van den
Heever J. P., Hutchinson C. R., Vederas J. C. (2000). Lovastatin Nonaketide
Synthase Catalyzes an Intramolecular Diels–Alder Reaction of
a Substrate Analogue. J. Am. Chem. Soc..

[9] Ose T., Watanabe K., Mie T., Honma M., Watanabe H., Yao M., Oikawa H., Tanaka I. (2003). Insight into a natural Diels–Alder
reaction from the structure of macrophomate synthase. Nature.

[10] Kim H. J., Ruszczycky M. W., Choi S.-h., Liu Y.-n., Liu H.-w. (2011). Enzyme-catalysed
[4 + 2] cycloaddition is a key step in the biosynthesis of spinosyn
A. Nature.

[11] Wang H., Zou Y., Li M., Tang Z., Wang J., Tian Z., Strassner N., Yang Q., Zheng Q., Guo Y., Liu W., Pan L., Houk K. N. (2023). A cyclase that catalyses competing
2 + 2 and 4 + 2 cycloadditions. Nat. Chem..

[12] Zhang X., King-Smith E., Renata H. (2018). Total Synthesis of Tambromycin by
Combining Chemocatalytic and Biocatalytic C–H Functionalization. Angew. Chem., Int. Ed..

[13] Lazzarotto M., Hammerer L., Hetmann M., Borg A., Schmermund L., Steiner L., Hartmann P., Belaj F., Kroutil W., Gruber K., Fuchs M. (2019). Chemoenzymatic Total Synthesis of
Deoxy-, epi-, and Podophyllotoxin and a Biocatalytic Kinetic Resolution
of Dibenzylbutyrolactones. Angew. Chem., Int.
Ed..

[14] Ortiz
de Montellano P. R. (2010). Hydrocarbon Hydroxylation by Cytochrome P450 Enzymes. Chem. Rev..

[15] Stout C. N., Renata H. (2021). Reinvigorating the Chiral Pool: Chemoenzymatic Approaches
to Complex Peptides and Terpenoids. Acc. Chem.
Res..

[16] Zwick C. R., Renata H. (2018). Remote C–H Hydroxylation by
an α-Ketoglutarate-Dependent Dioxygenase Enables Efficient Chemoenzymatic
Synthesis of Manzacidin C and Proline Analogs. J. Am. Chem. Soc..

[17] Chakrabarty S., Wang Y., Perkins J. C., Narayan A. R. H. (2020). Scalable biocatalytic
C–H oxyfunctionalization reactions. Chem.
Soc. Rev..

[18] Xiao H., Bao Z., Zhao H. (2015). High Throughput
Screening and Selection Methods for
Directed Enzyme Evolution. Ind. Eng. Chem. Res..

[19] Wang T.-C., Mai B. K., Zhang Z., Bo Z., Li J., Liu P., Yang Y. (2024). Stereoselective amino
acid synthesis by photobiocatalytic
oxidative coupling. Nature.

[20] Cheng L., Li D., Mai B. K., Bo Z., Cheng L., Liu P., Yang Y. (2023). Stereoselective amino
acid synthesis by synergistic photoredox-pyridoxal
radical biocatalysis. Science.

[21] Li M., Yuan Y., Harrison W., Zhang Z., Zhao H. (2024). Asymmetric
photoenzymatic incorporation of fluorinated motifs into olefins. Science.

[22] Harrison W., Jiang G., Zhang Z., Li M., Chen H., Zhao H. (2024). Photoenzymatic Asymmetric Hydroamination for Chiral Alkyl Amine Synthesis. J. Am. Chem. Soc..

[23] Xing Z., Liu F., Feng J., Yu L., Wu Z., Zhao B., Chen B., Ping H., Xu Y., Liu A., Zhao Y., Wang C., Wang B., Huang X. (2025). Synergistic
photobiocatalysis for enantioselective triple-radical sorting. Nature.

[24] Xu Y., Chen H., Yu L., Peng X., Zhang J., Xing Z., Bao Y., Liu A., Zhao Y., Tian C., Liang Y., Huang X. (2024). A light-driven enzymatic
enantioselective radical acylation. Nature.

[25] Biegasiewicz K. F., Cooper S. J., Gao X., Oblinsky D. G., Kim J. H., Garfinkle S. E., Joyce L. A., Sandoval B. A., Scholes G. D., Hyster T. K. (2019). Photoexcitation
of flavoenzymes enables a stereoselective
radical cyclization. Science.

[26] Reetz M. T., Becker M. H., Klein H.-W., Stöckigt D. (1999). A Method for
High-Throughput Screening of Enantioselective Catalysts. Angew. Chem., Int. Ed..

[27] Reetz M. T., Eipper A., Tielmann P., Mynott R. (2002). A Practical NMR-Based
High-Throughput Assay for Screening Enantioselective Catalysts and
Biocatalysts. Adv. Synth. Catal..

[28] Reetz M. T., Becker M. H., Kühling K. M., Holzwarth A. (1998). Time-Resolved
IR-Thermographic Detection and Screening of Enantioselectivity in
Catalytic Reactions. Angew. Chem., Int. Ed..

[29] Janes L. E., Kazlauskas R. J. (1997). QuickE.
A Fast Spectrophotometric Method To Measure
the Enantioselectivity of Hydrolases. J. Org.
Chem..

[30] Swoboda A., Pfeifenberger L. J., Duhović Z., Bürgler M., Oroz-Guinea I., Bangert K., Weißensteiner F., Parigger L., Ebner K., Glieder A., Kroutil W. (2023). Enantioselective
High-Throughput Assay Showcased for the Identification of (R)- as
well as (S)-Selective Unspecific Peroxygenases for C–H Oxidation. Angew. Chem., Int. Ed..

[31] Leung D., Kang S. O., Anslyn E. V. (2012). Rapid determination of enantiomeric
excess: a focus on optical approaches. Chem.
Soc. Rev..

[32] Jo H. H., Lin C.-Y., Anslyn E. V. (2014). Rapid Optical
Methods for Enantiomeric
Excess Analysis: From Enantioselective Indicator Displacement Assays
to Exciton-Coupled Circular Dichroism. Acc.
Chem. Res..

[33] Wolf C., Bentley K. W. (2013). Chirality sensing
using stereodynamic probes with distinct
electronic circular dichroism output. Chem.
Soc. Rev..

[34] Bentley K.
W., Zhang P., Wolf C. (2016). Miniature high-throughput chemosensing
of yield, ee, and absolute configuration from crude reaction mixtures. Sci. Adv..

[35] Hirose K., Fujiwara A., Matsunaga K., Aoki N., Tobe Y. (2002). Chiral recognition
of secondary amines by using chiral crown ether and podand. Tetrahedron Lett..

[36] Jiang L., Tian J., Zhao F., Yu S., Shi D., Wang X., Yu X., Pu L. (2019). Fluorescent Recognition
of Functional Secondary Amines in the Fluorous Phase. Eur. J. Org. Chem..

[37] Gu G., Zhao C., Zhang W., Weng J., Xu Z., Wu J., Xie Y., He X., Zhao Y. (2024). Chiral Discrimination
of Acyclic Secondary Amines by ^19^F NMR. Anal. Chem..

[38] Xu Z., Liu C., Zhao S., Chen S., Zhao Y. (2019). Molecular
Sensors for
NMR-Based Detection. Chem. Rev..

[39] Li Y., Wen L., Meng H., Lv J., Luo G., Zhao Y. (2020). Separation-free
Enantiodifferentiation with Chromatogram-like Output. Cell Rep. Phys. Sci..

[40] Xu Z., Wang C., He S., Wu J., Zhao Y. (2025). Enhancing
Molecular-Level Biological Monitoring with a Smart Self-Assembling ^19^F-Labeled Probe. Angew. Chem., Int.
Ed..

[41] Chen Y.-T., Li B., Chen J.-L., Su X.-C. (2022). Simultaneous Discrimination and Quantification
of Enantiomeric Amino Acids under Physiological Conditions by Chiral ^19^F NMR Tag. Anal. Chem..

[42] Wang W., Xia X., Bian G., Song L. (2019). A Chiral Sensor for Recognition of
Varied Amines Based on ^19^F NMR Signals of Newly Designed
Rhodium Complexes. Chem. Commun..

[43] Duong Q., Kwahk E.-J., Kim J., Park H., Cho H., Kim H. (2024). Bioinspired Fluorine
Labeling for ^19^F NMR-Based Plasma
Amine Profiling. Anal. Chem..

[44] Gu G., Xu Z., Wen L., Liang J., Wang C., Wan X., Zhao Y. (2023). Chirality
Sensing of N-Heterocycles via ^19^F NMR. JACS Au.

[45] France S. P., Howard R. M., Steflik J., Weise N. J., Mangas-Sanchez J., Montgomery S. L., Crook R., Kumar R., Turner N. J. (2018). Identification
of Novel Bacterial Members of the Imine Reductase Enzyme Family that
Perform Reductive Amination. ChemCatChem..

[46] Zhan Z. Z., Xu Z. F., Yu S. S., Feng J. H., Liu F. F., Yao P. Y., Wu Q. Q., Zhu D. M. (2022). Stereocomplementary
Synthesis of a Key Intermediate for Tofacitinib via Enzymatic Dynamic
Kinetic Resolution-Reductive Amination. Adv.
Synth. Catal..

[47] Aleku G. A., France S. P., Man H., Mangas-Sanchez J., Montgomery S. L., Sharma M., Leipold F., Hussain S., Grogan G., Turner N. J. (2017). A reductive aminase from Aspergillus
oryzae. Nat. Chem..

[48] Kumar R., Karmilowicz M. J., Burke D., Burns M., Clark L. A., Connor C. G., Cordi E., Do N. M., Doyle K. M., Hoagland S., Lewis C. A., Mangan D., Martinez C. A., McInturff E. L., Meldrum K., Pearson R., Steflik J., Rane A., Weaver J. (2021). Biocatalytic reductive amination
from discovery to commercial manufacturing applied to abrocitinib
JAK1 inhibitor. Nat. Catal..

[49] Thorpe T. W., Marshall J. R., Harawa V., Ruscoe R. E., Cuetos A., Finnigan J. D., Angelastro A., Heath R. S., Parmeggiani F., Charnock S. J., Howard R. M., Kumar R., Daniels D. S. B., Grogan G., Turner N. J. (2022). Multifunctional
biocatalyst for conjugate
reduction and reductive amination. Nature.

[50] Roiban G.-D., Kern M., Liu Z., Hyslop J., Tey P. L., Levine M. S., Jordan L. S., Brown K. K., Hadi T., Ihnken L. A. F., Brown M. J. B. (2017). Efficient
Biocatalytic Reductive
Aminations by Extending the Imine Reductase Toolbox. ChemCatChem..

[51] Ma E. J., Siirola E., Moore C., Kummer A., Stoeckli M., Faller M., Bouquet C., Eggimann F., Ligibel M., Huynh D., Cutler G., Siegrist L., Lewis R. A., Acker A. C., Freund E., Koch E., Vogel M., Schlingensiepen H., Oakeley E. J., Snajdrova R. (2021). Machine-Directed
Evolution of an Imine Reductase for Activity and Stereoselectivity. ACS Catal..

[52] Xu Z., Yao P., Sheng X., Li J., Li J., Yu S., Feng J., Wu Q., Zhu D. (2020). Biocatalytic Access
to 1,4-Diazepanes via Imine Reductase-Catalyzed Intramolecular Asymmetric
Reductive Amination. ACS Catal..

[53] Zhu J. M., Tan H. Q., Yang L., Dai Z., Zhu L., Ma H. M., Deng Z. X., Tian Z. H., Qu X. D. (2017). Enantioselective
Synthesis of 1-Aryl-Substituted Tetrahydroisoquinolines Employing
Imine Reductase. ACS Catal..

[54] Li H., Luan Z. J., Zheng G. W., Xu J. H. (2015). Efficient Synthesis
of Chiral Indolines using an Imine Reductase from Paenibacillus lactis. Adv. Synth. Catal..

[55] Leipold F., Hussain S., Ghislieri D., Turner N. J. (2013). Asymmetric Reduction
of Cyclic Imines Catalyzed by a Whole-Cell Biocatalyst Containing
an (S)-Imine Reductase. ChemCatChem..

[56] Zhang J., Liao D. H., Chen R. C., Zhu F. F., Ma Y. Q., Gao L., Qu G., Cui C. S., Sun Z. T., Lei X. G., Gao S. S. (2022). Tuning
an Imine Reductase for the Asymmetric Synthesis
of Azacycloalkylamines by Concise Structure-Guided Engineering. Angew. Chem., Int. Ed..

[57] Schober M., MacDermaid C., Ollis A. A., Chang S., Khan D., Hosford J., Latham J., Ihnken L. A. F., Brown M. J. B., Fuerst D., Sanganee M. J., Roiban G. D. (2019). Chiral synthesis
of LSD1 inhibitor GSK2879552 enabled by directed evolution of an imine
reductase. Nat. Catal..

[58] Wang Z., Gao G. S., Gao Y. D., Yang L. C. (2024). Application of Imine
Reductase in Bioactive Chiral Amine Synthesis. Org. Process Res. Dev..

[59] Aleku G. A. (2024). Imine Reductases
and Reductive Aminases in Organic Synthesis. ACS Catal..

[60] Citoler J., Harawa V., Marshall J. R., Bevinakatti H., Finnigan J. D., Charnock S. J., Turner N. J. (2021). Synthesis of Pharmaceutically
Relevant 2-Aminotetralin and 3-Aminochroman Derivatives via Enzymatic
Reductive Amination. Angew. Chem., Int. Ed..

[61] Tang D. Y., Ma Y. Q., Bao J. P., Gao S. S., Man S. L., Cui C. S. (2024). Chemoenzymatic total
synthesis of rotigotine *via* IRED-catalyzed reductive
amination. Org. Biomol. Chem..

[62] Reetz M. T., Wang L.-W., Bocola M. (2006). Directed Evolution
of Enantioselective
Enzymes: Iterative Cycles of CASTing for Probing Protein-Sequence
Space. Angew. Chem., Int. Ed..

[63] Reetz M. T., Carballeira J. D. (2007). Iterative
saturation mutagenesis (ISM) for rapid directed
evolution of functional enzymes. Nat. Protoc..

