# Supplementary material for: High-Throughput 19F NMR Chiral Analysis for Screening and Directed Evolution of Imine Reductases
Source: ACS Cent Sci. 2025 Jun 3;11(7):1094–102. doi: 10.1021/acscentsci.5c00498 (PMC12291108; doi:10.1021/acscentsci.5c00498)
Supplement: Supplementary file 1 [file oc5c00498_si_001.pdf]

Supporting Information

High-Throughput ^{19}F NMR Chiral Analysis for Screening and Directed Evolution of Imine Reductases

Shucheng Song,[1,‡] Chenyang Wang,[2,‡] Wenjing Bao,[2] Zhenchuang Xu,[2] Jian Wu,[3] Liang Lin,[1*] Yanchuan Zhao[2,3*]

[1]State Key Laboratory of Chemical Biology, Shanghai Institute of Organic Chemistry, University of Chinese Academy of Sciences, Chinese Academy of Sciences, 345 Ling-Ling Road, Shanghai 200032, China.

[2]State Key Laboratory of Fluorine and Nitrogen Chemistry and Advanced Materials and Shanghai Hongkong Joint Laboratory in Chemical Synthesis, Shanghai Institute of Organic Chemistry, University of Chinese Academy of Sciences, Chinese Academy of Sciences, 345 Ling-Ling Road, Shanghai 200032, China.

[3]Instrumental Analysis Center, Shanghai Institute of Organic Chemistry, Chinese Academy of Sciences, Shanghai, 200032, China.

‡These authors contributed equally.

*Correspondence: zhaoyanchuan@sioc.ac.cn; lianglin@mail.sioc.ac.cn.

Table of Contents

Materials and Methods S2

DNA Sequence and Amino acid sequence of IRED-195 .... S8

Preparation of **probe-CF_3** S9

Evaluation of Enantiomeric Excess Values and Yields Assessment of Product **1a** S10

Screening of Yields and Enantiomeric Excess Values for Product **1a** via ^{19}F NMR Analysis S13

Evaluation of Enantiomeric Excess Values and Yields Assessment of Product **2a** S17

Screening of Yields and Enantiomeric Excess values of Product **2a** in Directed Evolution via ^{19}F NMR Analysis S21

Evaluation of Enantiomeric Excess Values and Yields Assessment of Product **4a** S24

Screening of Yields and Enantiomeric Excess Values of Product **4a** via ^{19}F NMR Analysis S28

Sequence Alignment Stereoselective IREDs S32

Structural Overlays and Active Site Comparisons of IREDs S33

A Comparison Between Enantiomeric Excess Values **1a** Determined by ^{19}F NMR and Chiral HPLC Analysis S37

Exemplified High Throughput Screening of Enantiocomposition of **1a** via ^{19}F NMR S38

Validation of the ^{19}F NMR Platform in Whole-Cell Based Systems S39

Performance of WT and Mutants of IRED-195 .... S39

Primers Used for Site-Saturation Mutagenesis of 9 Sites S40

Sequence IDs of the Panel of 134 IREDs S45

References S47

^{1}H and ^{19}F NMR Spectra of **probe-CF_3** S48

Materials and Methods

Materials:

All commercial chemicals and reagents, including substrates **1**, **2**, **4**, amine **3** and β-nicotinamide adenine dinucleotide phosphate ($NADP^+$) were purchased from Energy Chemical, Bontac Bio-engineering (Shenzhen, China), Bide Pharmatech (Shanghai, China), Macklin (Shanghai, China), Haohong scientific (Shanghai, China), Adamas (Shanghai, China) or Innochem (Beijing, China) unless otherwise specified. HPLC-grade solvents (*n*-hexane and *i*-propanol) were purchased from Thermo Fisher Scientific (Waltham, Massachusetts, USA). Potassium phosphate buffer solutions (0.1 M, pH = 7.0 or pH = 9.0) was prepared using ultrapure water (18.2 MΩ·cm) in our laboratory. **Probe-CF_3** was synthesized according to the procedure described in the referenced literature.[1]

Methods for ^{19}F NMR Spectroscopy:

^{1}H and ^{19}F NMR spectra for products characterization were recorded by Agilent-400, Bruker Avance-400, or Bruker Avance-600 spectrometer, chemical shifts (δ) are reported in parts per million (ppm) and referenced with TMS or solvent residue for ^{1}H NMR, and $CFCl_3$ for ^{19}F NMR. The solutions used for analysis were prepared by mixing the probe and analyte in $CDCl_3$. For Figures 2B, 2C, 3B, 3C, S1, S3, S12, S14, ^{19}F NMR spectra were recorded on a Bruker Avance neo 600 NMR spectrometer (565 MHz for ^{19}F nucleus) using a scan number of 16. For Figures 4D, S6, S8, ^{19}F NMR spectra were recorded on a Bruker Avance neo 600 NMR spectrometer (565 MHz for ^{19}F nucleus) using a scan number of 64.

Methods for protein expression:

Plasmids carrying the genes encoding the target enzymes were transformed into *E. coli* BL21(DE3) competent cells to facilitate protein production. These cells were initially cultured in 300 µL of LB medium supplemented with 50 µg/mL kanamycin and incubated overnight at 37 °C with agitation at 700 rpm. The following day, 20 µL of this pre-culture was used to inoculate 1 mL of Terrific Broth (TB) medium. This culture was then incubated at 37 °C with continuous shaking at 700 rpm until the optical density at 600 nm (OD600) reached between 0.6 and 0.8, indicating adequate cell growth. Protein expression was subsequently initiated by adding 0.3 mM isopropyl-β-D-thiogalactopyranoside (IPTG), and the culture was incubated for an additional 24 hours at 20 °C with increased shaking at 1000 rpm to enhance protein folding and stability. The cells were then collected by centrifugation at 4000 rpm for 15 minutes, and the pellet was washed with 100 mM potassium phosphate buffer (pH = 7.0) to eliminate any remaining TB medium. A second centrifugation at the same speed and duration was performed before resuspending the cells in the reaction buffer. Cells were disrupted through three freeze-thaw cycles using liquid nitrogen and a subsequent thawing on ice. To support enzymatic reactions, 1 mg/mL glucose dehydrogenase (GDH) was added to the system, which was then incubated at 30 °C with shaking at 700 rpm to facilitate the enzymatic activity.

Methods for site-saturation mutagenesis library construction:

Site-saturation mutagenesis libraries were created using the overlap extension PCR technique, with recombinant plasmids pET28a carrying the IRED-195 gene serving as the template. The PCR protocol commenced with an initial denaturation at 98 °C for 30 seconds, followed by 19 cycles of denaturation at

98 °C for 10 seconds, annealing at the primer-specific temperature for 30 seconds, and elongation at 72 °C for 4 minutes. The program concluded with a final extension sequence involving a denaturation at 98 °C for 10 seconds, primer annealing for 30 seconds, elongation at 72 °C for 4 minutes, annealing for the overlap at the specific temperature for 1 minute, and a final elongation at 72 °C for 4 minutes. The PCR reaction mixture was composed of 0.5 µL of each forward and reverse primer (10 mM), 0.1 µL NEB Q5 polymerase, 1 ng of the plasmid template, 0.4 µL dNTPs (5 mM), 2 µL of 5× Q5 Reaction Buffer, and 5.5 µL of distilled water, totaling 10 µL. After amplification, the PCR products underwent Dpn I digestion for 1 hour at 37 °C to eliminate methylated, non-mutated parental DNA. The mutagenesis library was subsequently transformed into *E. coli* BL21(DE3) competent cells for further experimentation.

High-Throughput Screening Methods for 134 Imine Reductases (IREDs) in the Synthesis of 1a and 4a:
The typical reaction involved a 300 µL mixture containing 0.5 mM $NADP^+$, 20 mM of either substrate **1** or **4**, 30 mM D-glucose, and 1 mg/mL GDH in a 100 mM potassium phosphate buffer (pH = 9.0). These reactions were incubated at 30 °C with agitation at 800 rpm for 12 hours. Post-incubation, the reactions were terminated by adding 5 µL of 5 M sodium carbonate. For analytical purposes, an internal standard of 2 µmol *R*-2-methylpiperidine (**Int-I**) was added. The resulting mixture was then extracted with 500 µL of deuterated chloroform, which was enhanced with 8 mg/mL of **probe-CF_3** to aid in NMR analysis. The organic phase was subsequently subjected to ^1H-decoupled ^{19}F-NMR, conducting 16 scans to evaluate both the yield and the enantiomeric excess (*ee*) of the reactions. This methodology provides a detailed assessment of each IRED's catalytic capabilities towards the imine reduction of the specified substrates.

High-Throughput Screening Methods for Biocatalytic Synthesis of 2a:
For high-throughput screening of the biocatalytic reaction to produce compound **2a**, gene expression was initiated in 96-well plates. Colonies were selected and inoculated into deep-well plates filled with 300 µL of LB medium enriched with 50 µg/mL kanamycin. These cultures were incubated overnight at 37 °C with shaking at 800 rpm. Subsequently, a 20 µL aliquot from each culture was transferred to a new plate containing 1 mL of Terrific Broth (TB) medium with 50 µg/mL kanamycin, and incubated under the same conditions until the optical density at 600 nm (OD600) reached 0.6–0.8. Protein expression was triggered by adding 0.3 mM IPTG, followed by a 24-hour incubation at 20 °C. The cells were then harvested by centrifugation at 4000 rpm for 15 minutes, and washed with 200 µL of 100 mM potassium phosphate buffer (pH = 7.0). For cell lysate, the cell pellets were resuspended in 500 µL of the same buffer supplemented with BugBuster and 6 U of DNase I, and incubated at 30 °C for 2 hours with shaking. Then the cell debris was removed by centrifugation, and 200 µL of the clear supernatant was mixed with 300 µL of a reaction stock solution in new deep-well plates. This solution contained 0.5 mM $NADP^+$, 10 mM of substrate **2**, 1 M amine **3** (100 equivalents), 30 mM *D*-glucose, 1 mg/mL GDH, and 20% v/v DMSO. The reaction mixture was incubated at 30 °C with shaking at 800 rpm for 12 hours. Reactions were terminated by adding 50 µL of 5 M NaOH. For analysis, 1 µmol of (*R*)-1-methyl-1,2,3,4-tetrahydroisoquinoline (**Int-II**) was added as an internal standard. The mixture was then extracted thrice with 300 µL ethyl acetate, and the organic phases were pooled and concentrated under reduced pressure to remove excess amine **3**. For final analysis, 500 µL of deuterated chloroform containing 8 mg/mL **probe-CF_3** was added. Samples were analyzed using ^1H-decoupled ^{19}F-NMR, performing 64 scans to determine the reaction yield and *ee* values.

Methods for generating and screening iterative saturation mutagenesis libraries

Library Construction:

Key residues governing stereoselectivity and activity were identified from initial saturation mutagenesis libraries (Supplementary Table S3). Iterative saturation mutagenesis libraries were created using the overlap extension PCR technique, with recombinant plasmids pET28a carrying the IRED-195 gene serving as the template. After amplification, the PCR products underwent Dpn I digestion for 1 hour at 37 °C to eliminate methylated, non-mutated parental DNA. The mutagenesis library was subsequently transformed into *E. coli* BL21(DE3) competent cells for further experimentation.

Library Screening:

High-throughput screening was done in 96-well plates using the same procedure as site-saturation mutagenesis. The 96-deep-well plates were added 200 μL of the supernatant of the cell lysate and 300 μL stock solution containing $NADP^+$ (1 mM), substrate **2** (50 mM), amine **3** (0.5 M), D-glucose (150 mM), BmGDH (1 mg/mL), potassium phosphate buffer (100 mM, pH = 7.0) and DMSO (20%, v/v). The reaction mixture was incubated at 30 °C with shaking at 800 rpm for 12 hours. Reactions were terminated by adding 50 μL of 5 M NaOH, followed by a five-fold dilution. For analysis, 1 μmol of (*R*)-1-methyl-1,2,3,4-tetrahydroisoquinoline (**Int-II**) was added as an internal standard. The mixture was then extracted thrice with 300 μL ethyl acetate, and the organic phases were pooled and concentrated under reduced pressure to remove excess amine **3**. For final analysis, 500 μL of deuterated chloroform containing 8 mg/mL **probe-CF_3** was added. Samples were analyzed using ^{1}H-decoupled ^{19}F-NMR, performing 64 scans to determine the reaction yield and *ee* values.

Methods for molecular docking:

Molecular docking simulations were carried out using AutoDock Vina software to predict the interactions between enzyme and substrate. During the docking setup, the enzyme residues were treated as rigid structures, while the substrate ligand was allowed flexibility to explore various binding conformations. We utilized a grid box of dimensions 22 × 22 × 22 Å, with a grid spacing of 1.0 Å, centered at coordinates (-14.93, -22.264, -4.334), strategically positioned to cover the substrate binding site. All other parameters were set to default to ensure consistent conditions for flexible docking. Multiple docking runs were executed under the program's standard settings, and the conformation displaying the lowest binding energy was selected for detailed analysis. This chosen conformation was scrutinized particularly for the interactions between the imine carbon of the substrate and the C4N atom of NADPH. The most promising substrate conformation, characterized by a pro-S near attack pose in the active site, was extracted and subsequently analyzed using PyMOL software for a deeper insight into the molecular interactions within the complex.

Procedure for the construction of standard curves for determination of yields and *ee* values of products 1a and 4a

Standard curve for quantifying the yields of products 1a and 4a:

To establish a standard curve for quantifying the yields of products **1a** and **4a**, a series of calibration samples were prepared within a total reaction volume of 300 μL. For each product, five samples were prepared, containing incremental amounts of racemic 2-methylpyrrolidine or racemic 1-methyl-1,2,3,4-

tetrahydroisoquinoline at concentrations of 1.2 μmol, 2.4 μmol, 3.6 μmol, 4.8 μmol, and 6.0 μmol. Each sample also included (*R*)-2-methylpiperidine at 2 μmol as an internal standard to facilitate accurate quantification. These samples were dissolved in 100 mM potassium phosphate buffer (pH = 9.0). Following sample preparation, 5 μL of 5 M NaOH was added to neutralize the mixtures, which were then extracted with 500 μL of deuterated chloroform containing 8 mg/mL **probe-CF_3**. The resulting organic extracts were analyzed using ^{1}H-decoupled ^{19}F NMR, with each sample undergoing 16 scans. This method was used to accurately quantify the concentrations of products **1a** and **4a** in the reaction mixtures.

Standard curve for determination of *ee* values of products 1a and 4a:

To establish a standard curve for determining the *ee* values of products **1a** and **4a**, a calibration protocol was developed with a reaction volume of 300 μL for each sample. A total of 21 samples were prepared, encompassing a range of molar ratios between the (*R*)- and (*S*)-enantiomers of 2-methylpyrrolidine and 1-methyl-1,2,3,4-tetrahydroisoquinoline. These molar ratios varied from 20 mM to 0 mM for each enantiomer, representing *ee* values (%) from -99% to +99% in increments of 10%. Specifically, the *ee* values were arranged as: 99 (S), 90 (S), 80 (S), 70 (S), 60 (S), 50 (S), 40 (S), 30 (S), 20 (S), 10 (S), 0 (racemic), 10 (R), 20 (R), 30 (R), 40 (R), 50 (R), 60 (R), 70 (R), 80 (R), 90 (R), and 99 (R). Each sample was dissolved in 100 mM potassium phosphate buffer (pH = 9.0) to ensure consistent solubility and reaction conditions. After preparation, each sample was neutralized by adding 5 μL of 5 M NaOH, followed by extraction with 500 μL of deuterated chloroform containing 8 mg/mL **probe-CF_3** to facilitate NMR analysis. The organic extracts were then analyzed using ^{1}H-decoupled ^{19}F NMR, with each sample undergoing 16 scans. This analytical setup provided precise quantification of the *ee* values for products **1a** and **4a**, essential for evaluating the stereoselectivity of the biocatalytic processes involved.

Standard curves for determine the yields and *ee* values of product 2a:

For product **2a**, standard curves were established to determine both the yields and *ee* values using a total reaction volume of 500 μL. Four individual samples were prepared, each containing varying amounts of racemic 2-(N-propylamino)-5-methoxytetralin (2.0 μmol, 3.0 μmol, 4.0 μmol, and 5.0 μmol). Additionally, each sample included 500 μmol of amine **3** and 10 μmol of (*R*)-1-methyl-1,2,3,4-tetrahydroisoquinol (**Int-II**) as an internal standard. These components were dissolved in 100 mM potassium phosphate buffer (pH = 7.0) to ensure optimal reaction conditions. Following the preparation, 5 μL of 5 M NaOH was added to each mixture to neutralize any acidic components. The samples were then extracted three times with 300 μL of ethyl acetate. The combined organic phases were concentrated under reduced pressure to effectively remove excess amine **3**. For NMR analysis, 500 μL of deuterated chloroform containing 8 mg/mL **probe-CF_3** was added to the concentrated extracts. The prepared samples underwent analysis using ^{1}H-decoupled ^{19}F-NMR, performing 64 scans to accurately determine both the yield and the *ee* values of product **2a**.

Standard curve for determination of ee values of products 2a:

To establish a standard curve for determining the enantiomeric excess values of products **2a**, a calibration protocol was developed. A series of 10 samples were prepared in a reaction volume of 500 μL each, covering a range of molar ratios between the (*rac*)- and (*S*)-enantiomers of 2-(*N*-propylamino)-5-methoxytetralin. The molar ratios for each component ranged from 10 mM to 0 mM, corresponding to *ee* values (%) from 0 to 95

in increments of 10%. Specifically, the *ee* values were systematically arranged as follows: 95 (S), 90 (S), 80 (S), 70 (S), 60 (S), 50 (S), 40 (S), 30 (S), 20 (S), 10 (S), and 0 (racemic mixture). Each sample was dissolved in 500 µL of deuterated chloroform containing 8 mg/mL of **probe-CF_3** to facilitate NMR analysis. All samples were subjected to ^{1}H-decoupled ^{19}F NMR analysis, with each spectrum acquired using 64 scans.

Procedure for comparison between Chiral HPLC analysis and ^{19}F NMR for product 1a

Biological experiments: The reaction mixture (600 µL) consisted of 0.5 mM $NADP^+$, 20 mM substrate **1**, 30 mM *D*-glucose, and 1 mg/mL GDH in 100 mM potassium phosphate buffer (pH = 9.0). The reactions were incubated at 30 °C with continuous agitation at 800 rpm for 12 hours. After incubation, the reactions were quenched by the addition of 5 µL of 5 M NaOH. The resulting mixture was extracted with 1 mL of $CDCl_3$.

Derivatization methods: For chiral HPLC analysis, 200 µL of the supernatant was transferred to a fresh tube and treated with 20 µL of 0.2 M triethylamine and 20 µL of 0.4 M benzoyl chloride. The mixture was shaken at room temperature for 2 hours to facilitate derivatization. Following derivatization, the sample was dried over anhydrous sodium sulfate, and the solvent was removed under reduced pressure. The residue was dissolved in a solvent mixture of *n*-hexane and *i*-propanol (90:10, v/v), filtered, and subjected to chiral HPLC analysis to determine the enantiomeric excess.

Chiral HPLC method: CHIRALPAK IC column with a mobile phase of *n*-Hexane/*i*-Propanol (90:10, v/v), flow rate 1.0 mL/min, 30 °C, UV detection at 254 nm.

^{19}F NMR analysis: the remaining supernatant was transferred to an NMR tube containing 4 mg of **probe-CF_3**. The sample was analyzed by ^{1}H-decoupled ^{19}F NMR without shimming, with 16 scans performed to assess the enantiomeric excess of the reaction products.

Procedure for validation of the ^{19}F NMR platform in whole-cell and cell lysate biocatalytic systems:

Plasmids carrying the genes encoding the target enzymes were transformed into *E. coli* BL21(DE3) competent cells to facilitate protein production. These cells were initially cultured in 5 mL of LB medium supplemented with 50 µg/mL kanamycin and incubated overnight at 37 °C with agitation at 700 rpm. The following day, 400 µL of this pre-culture was used to inoculate 25 mL of Terrific Broth (TB) medium. This culture was then incubated at 37 °C with continuous shaking at 700 rpm until the optical density at 600 nm (OD600) reached between 0.6 and 0.8, indicating adequate cell growth. Protein expression was subsequently initiated by adding 0.3 mM isopropyl-β-D-thiogalactopyranoside (IPTG), and the culture was incubated for an additional 24 hours at 20 °C with increased shaking at 220 rpm to enhance protein folding and stability. The cells were then collected by centrifugation at 8000 rpm for 15 minutes, and the pellet was washed with 100 mM potassium phosphate buffer (pH = 7.0) to eliminate any remaining TB medium. A second centrifugation at the same speed and duration was performed before resuspending the cells in the reaction buffer.

Whole-cell system: *E. coli* cells were harvested as a wet cell pellet and resuspended in pH 9.0 100 mM potassium phosphate buffer (1 g wet cell weight per 5 mL buffer)

Cell lysate: The pellet was resuspended in pH 9.0 100 mM potassium phosphate buffer (1 g wet cell weight per 5 mL buffer) and lysed by sonication (70% amplitude, 3 × 10 s pulses) and the supernatant was adjusted to a total protein concentration of 10 mg/mL.

Procedure for preparative scale biotransformation of 2a

Biotransformation was performed on a 2.5 mmol scale in a 50 mL reaction mixture containing substrate **2** (50 mM), amine **3** (0.5 M), D-glucose (150 mM), lyophilized IRED-195-R2 lysate (1 mg/mL), $NADP^+$ (2.5 mM), BmGDH (1 mg/mL), and potassium phosphate buffer (100 mM, pH 7.0) in 20% (v/v) DMSO. The reaction mixture was adjusted to 7.0 with 1 M HCl or NaOH, and was incubated at 30 °C with shaking (220 rpm). After 24 h, 100 µL of the reaction mixture was collected and diluted five-fold and 50 µL of 5 M NaOH was added to the mixture. For analysis, 1 µmol of (*R*)-1-methyl-1,2,3,4-tetrahydroisoquinoline (**Int-II**) was added as an internal standard. The mixture was then extracted thrice with 300 µL ethyl acetate, and the organic phases were pooled and concentrated under reduced pressure to remove excess amine **3**. For final analysis, 500 µL of deuterated chloroform containing 8 mg/mL **probe-CF$_3$** was added. Samples were analyzed using ^{1}H-decoupled ^{19}F-NMR, performing 64 scans to determine the reaction yield and *ee* values.

DNA Sequence and Amino acid sequence of IRED-195

DNA sequence of IRED-195-WT

ATGCCAGAAAGCACCACTCCGTCTACTGCAACTCCAGTTACCATCATTGGTCTGGGTGCAATGGG
TACTGCTCTGGCAAACGCATTCCTGGATGCAGGTCATAGCACCACCGTTTGGAACCGTACTGCTG
CACGTGCAACTGCACTGGCAGCTCGTGGTGCACATCATGCAGAAACCGTTACTGAAGCGATTGC
AGCTTCTCCGCTGGTTATCGCATGTGTTCTGGATTACGATGCGTTCCACGAAACTCTGGCACCAG
CTACTGATGCACTGGCAGGTCGTGCACTGGTTAACCTGACCACTGGTACTCCGAAACAGGCTCG
TGAAACTGCATCTTGGGCTGCAGATCATCGTATCGATTACCTGGATGGCAAAATCATGGCAATTCC
ACCAGGTATCGCAACTCCAGATAGCTTCATCCTGTACTCTGGTCCACTGGGTACCTTCGAAGCAC
ATCGTTCTACCCTGGAAGTTCTGGGTGCAGCTAACCACGTTGGCACTGATGCAGGTCTGGCATCT
CTGCACGATATCGCACTGCTGACTGGTATGTATGGCATGATTGCAGGTATCCTGCAGGCATTCGCT
CTGATCGATTCTGAAGGTATTCCAGCAGGTGATCTGGCACCAATGCTGACCAACTGGCTGACTGG
TGCAGCTCATTCTGTTGCACATTACGCACAGCAGATTGATACTGGTGATTACGAAACCGGTGTTG
TTTTCAACCTGGCACATCAGTCTCATGGTTTTGCGAAACTGGTTCAGGCTGGTGAAGATCAGGGT
GTTGATGTTGGTCTGCTGCGTCCACTGTTCGAACTGATGCGTCATCAGGTTGCTGCAGGTTACGG
TAACGGTGATGTTGCGTCTGTTATCGAACTGATTCGTCGTGAAGAACGTCGTCAGCCAGCAAAAT
CTCCAGGTGCAGACAAAATCACTCGTGCACGTCGTCCA

Amino acid sequence of IRED-195-WT

MPESTTPSTATPVTIIGLGAMGTALANAFLDAGHSTTVWNRTAARATALAARGAHHAETVTEAIAAS
PLVIACVLDYDAFHETLAPATDALAGRALVNLTTGTPKQARETASWAADHRIDYLDGKIMAIPPGIAT
PDSFILYSGPLGTFEAHRSTLEVLGAANHVGTDAGLASLHDIALLTGMYGMIAGILQAFALIDSEGIPA
GDLAPMLTNWLTGAAHSVAHYAQQIDTGDYETGVVFNLAHQSHGFAKLVQAGEDQGVDVGLLRP
LFELMRHQVAAGYGNGDVASVIELIRREERRQPAKSPGADKITRARRP

Amino acid sequence of IRED-195-R2

MPESTTPSTATPVTIIGLGAMGTALANAFLDAGHSTTVWNRTAARATALAARGAHHAETVTEAIAAS
PLVIACVLDYDAFHETLAPATDALAGRALVNLTSGTPKQARETASWAADHRIDYLDGKIMAFPPGIA
TPDSFILYSGPLGTFEAHRSTLEVLGAANHVGTDAGLASLHDIALLTGMYGMIAGILQAFALIDSEGIP
AGDLAPMLTNWLTGMAHSVAHYAQQIDTGDYETGVVFNLAHQSHGNAKIVQAGEDQGVDVGLLR
PLFELIRHQVAAGYGNGDVASVIELIRREERRQPAKSPGADKITRARRP

Preparation of probe-CF_3

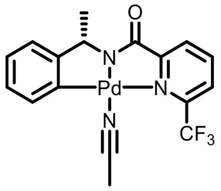

Probe-CF_3

Probe-CF_3 was synthesized following referenced literature.[1] ^{1}H NMR (400 MHz, Chloroform-*d*) δ 8.42 (d, *J* = 7.9 Hz, 1H), 8.09 (t, *J* = 7.8 Hz, 1H), 7.86 (d, *J* = 7.7 Hz, 1H), 7.11 – 6.97 (m, 2H), 6.97 – 6.68 (m, 2H), 5.16 (d, *J* = 6.7 Hz, 1H), 1.53 (d, *J* = 6.4 Hz, 3H).^{19}F NMR (376 MHz, Chloroform-*d*) δ -65.09 (s, 3F). The characterization data are consistent with those reported in the literature.[1]

Evaluation of Enantiomeric Excess Values and Yields Assessment of Product 1a

a) Establishment of the method to determine the *ee* values of **1a** via ^{19}F NMR

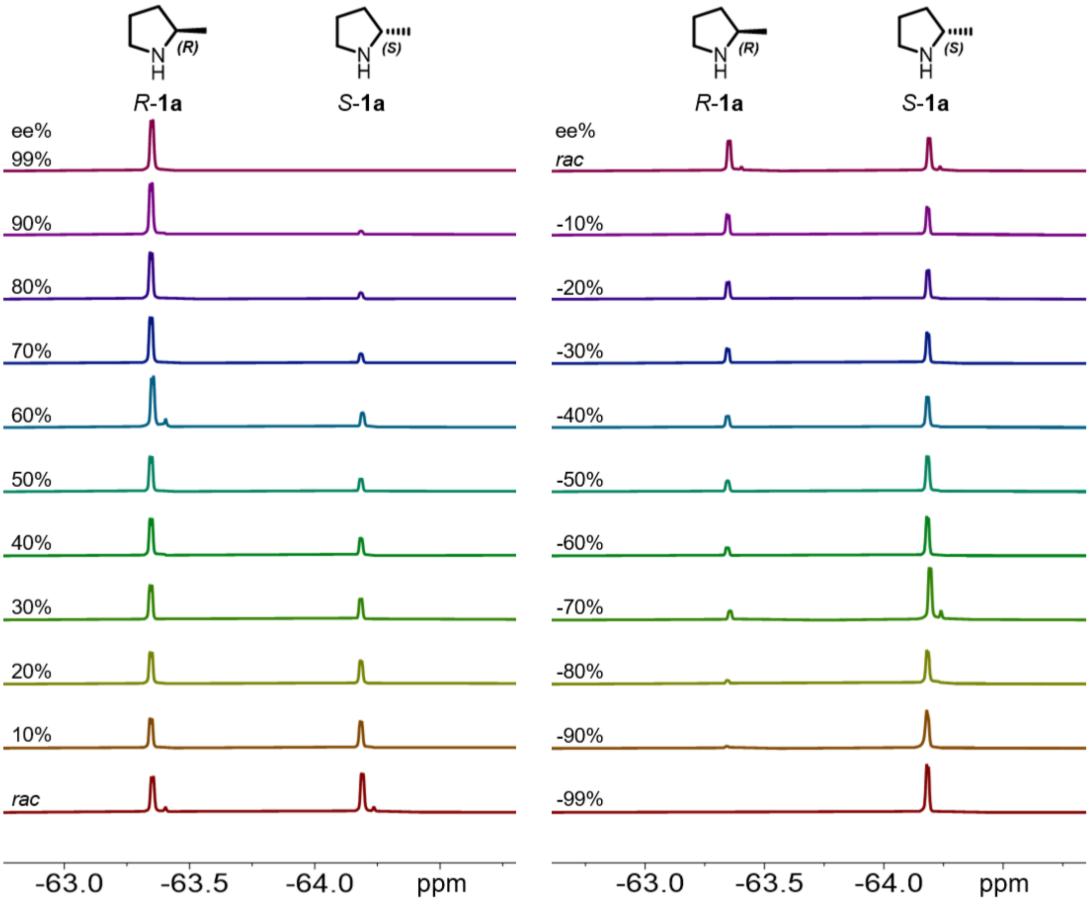

Figure S1. Evaluation of the *ee* values of **1a**. ^{1}H-decoupled ^{19}F NMR spectra of a mixture **probe-CF$_3$** (9 µmol), 2-methylpyrrolidine (ca. 6 µmol) of varying *ee* values in $CDCl_3$. ^{19}F NMR Spectra were recorded on a Bruker Avance-600 NMR spectrometer (565 MHz for the ^{19}F nucleus) using a scan number of 16.

b) Exemplified illustration of the calculation of *ee* values of **1a** via ^{19}F NMR

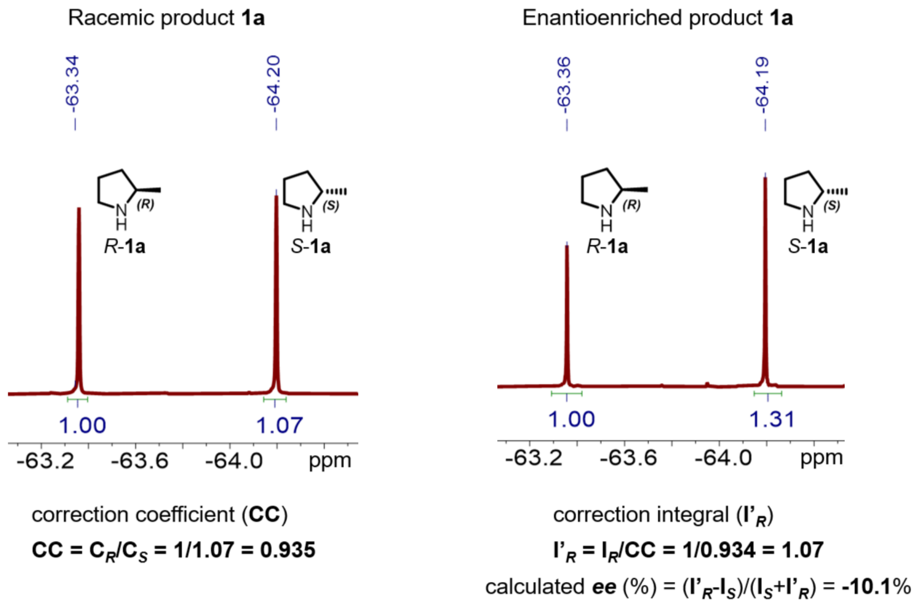

Figure S2. Evaluation of enantiomeric excess values of product **1a**. ^{19}F NMR Spectra were recorded on a Bruker Avance-600 NMR spectrometer (565 MHz for the ^{19}F nucleus) using a scan number of 16.

c) Establishment of the method to determine the yields of **1a** via ^{19}F NMR

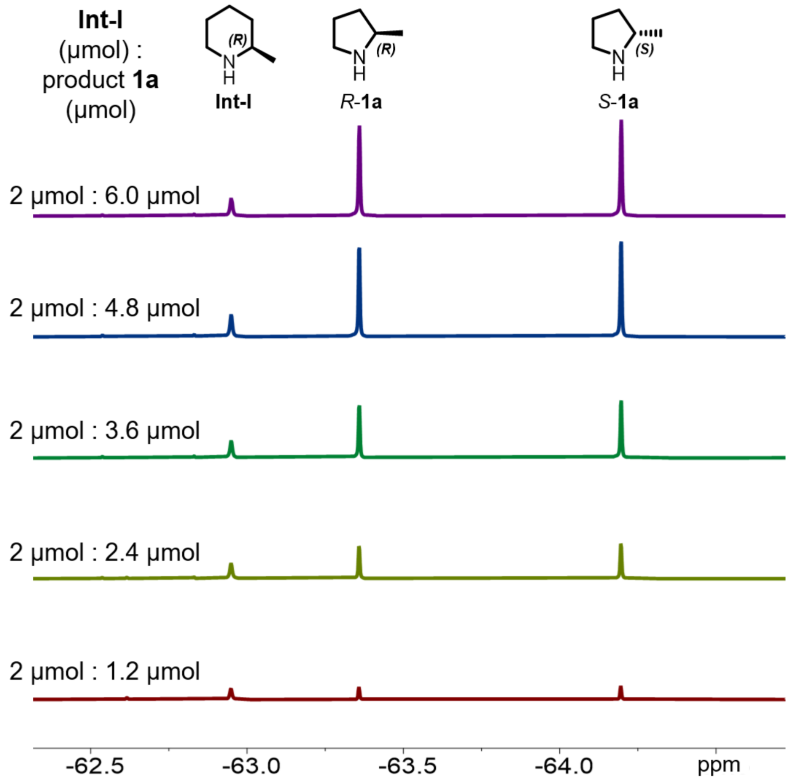

Figure S3. Establishment of the method to determine the yields of **1a**. ^{1}H-decoupled ^{19}F NMR spectra of a mixture of **probe-CF$_3$** (ca. 9 μmol), varying amounts of product **1a** (0 to 6 μmol) and 2 μmol of internal standard (*R*)-2-methylpiperidine (**Int-I**) in $CDCl_3$. ^{19}F NMR Spectra were recorded on a Bruker Avance-600 NMR spectrometer (565 MHz for the ^{19}F nucleus) using a scan number of 16.

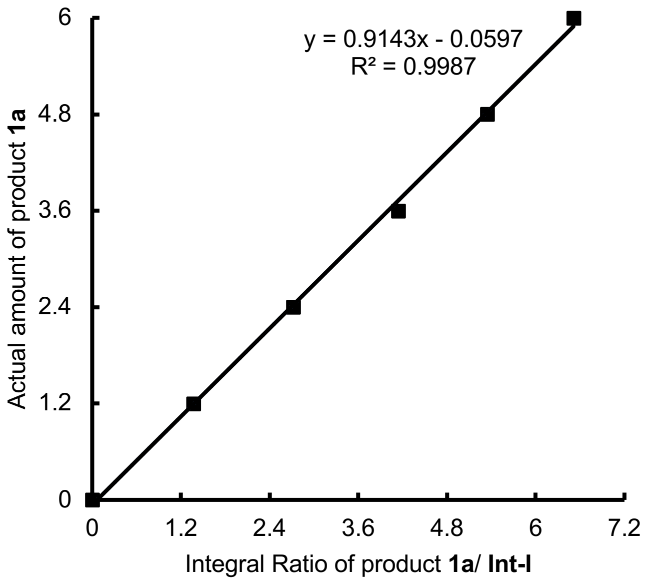

Figure S4. Linear correlation between ^{19}F NMR integral ratios and actual amounts of product **1a**

d) Exemplified illustration of the calculation of yields of **1a** via ^{19}F NMR

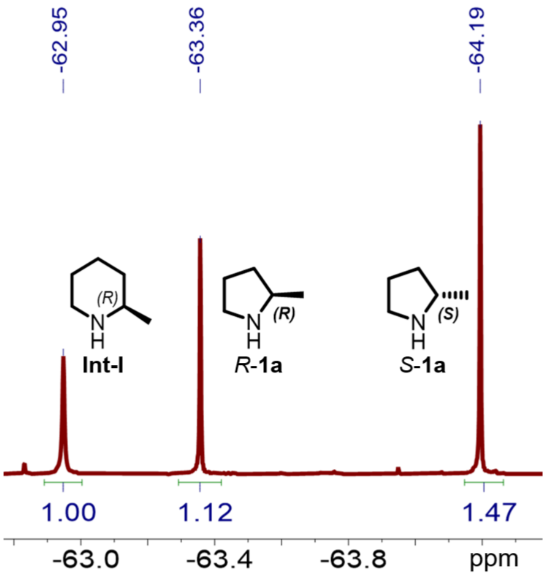

calculated **amount** (μmol) = 0.9143 × ($\mathbf{I}_R$ + $\mathbf{I}_S$) -0.0597 = **2.3 μmol**

calculated **yield**(%) = calculated amount/ theoretical amount ×100% = **38.3**%

Figure S5. Evaluation of the yield for product **1a**. ^{19}F NMR Spectra were recorded on a Bruker Avance-600 NMR spectrometer (565 MHz for the ^{19}F nucleus) using a scan number of 16.

Screening of Yields and Enantiomeric Excess Values for Product 1a via ^{19}F NMR Analysis

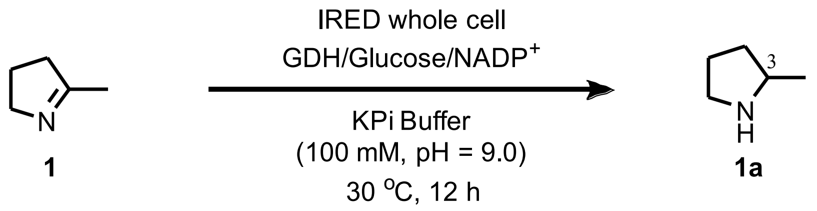

Table S1. Yields and *ee* values of **1a** determined by ^{19}F NMR Analysis

Enzyme	Intergral area of (R)-**1a**	Intergral area of (S)-**1a**	Integral area of **Int-I**	Corrected integral area of (S)-**1a**	Yield (%)	*ee* (%)
IRED-2	0.00	0.00	1	0.00	N.D.	N.D.
IRED-3	1.78	0.00	1	0.00	26	99(R)
IRED-5	0.00	0.00	1	0.00	N.D.	N.D.
IRED-6	4.74	0.12	1	0.11	73	95(R)
IRED-8	3.33	0.08	1	0.07	51	95(R)
IRED-12	0.00	0.00	1	0.00	N.D.	N.D.
IRED-13	0.00	0.00	1	0.00	N.D.	N.D.
IRED-17	0.00	0.00	1	0.00	N.D.	N.D.
IRED-18	3.08	0.13	1	0.12	48	92(R)
IRED-19	2.17	0.00	1	0.00	32	99(R)
IRED-20	2.18	0.11	1	0.10	34	91(R)
IRED-22	0.00	0.00	1	0.00	N.D.	N.D.
IRED-23	0.00	0.00	1	0.00	N.D.	N.D.
IRED-24	0.00	0.00	1	0.00	N.D.	N.D.
IRED-25	2.25	0.04	1	0.04	34	97(R)
IRED-27	0.00	0.00	1	0.00	N.D.	N.D.
IRED-28	3.28	0.06	1	0.06	50	96(R)
IRED-31	2.71	0.10	1	0.09	42	93(R)
IRED-32	0.00	0.00	1	0.00	N.D.	N.D.
IRED-33	0.00	0.00	1	0.00	N.D.	N.D.
IRED-34	3.15	0.09	1	0.08	48	94(R)
IRED-36	2.58	0.00	1	0.00	38	99(R)
IRED-37	0.00	0.00	1	0.00	N.D.	N.D.
IRED-42	0.00	0.00	1	0.00	N.D.	N.D.
IRED-44	0.00	0.00	1	0.00	N.D.	N.D.
IRED-45	0.00	0.00	1	0.00	N.D.	N.D.
IRED-47	0.00	0.00	1	0.00	N.D.	N.D.
IRED-48	0.00	0.00	1	0.00	N.D.	N.D.
IRED-49	0.00	0.00	1	0.00	N.D.	N.D.

IRED-50	2.54	0.07	1	0.07	39	95(R)
IRED-51	2.44	0.04	1	0.04	37	97(R)
IRED-52	0.00	0.00	1	0.00	N.D.	N.D.
IRED-54	0.00	0.00	1	0.00	N.D.	N.D.
IRED-55	3.66	0.13	1	0.12	57	94(R)
IRED-56	4.42	0.13	1	0.12	68	95(R)
IRED-59	0.00	0.00	1	0.00	N.D.	N.D.
IRED-60	1.54	0.08	1	0.07	24	90(R)
IRED-61	0.00	0.00	1	0.00	N.D.	N.D.
IRED-64	0.44	0.00	1	0.00	6	99(R)
IRED-66	1.75	0.09	1	0.08	27	91(R)
IRED-67	2.13	0.00	1	0.00	31	99(R)
IRED-68	0.00	0.00	1	0.00	N.D.	N.D.
IRED-69	3.36	0.10	1	0.09	52	95(R)
IRED-70	0.00	0.00	1	0.00	N.D.	N.D.
IRED-72	2.72	0.00	1	0.00	40	99(R)
IRED-73	4.11	0.17	1	0.16	64	93(R)
IRED-74	0.00	0.00	1	0.00	N.D.	N.D.
IRED-76	2.36	0.10	1	0.09	36	92(R)
IRED-81	2.86	0.00	1	0.00	43	99(R)
IRED-83	5.26	0.00	1	0.00	79	99(R)
IRED-84	1.03	0.00	1	0.00	15	99(R)
IRED-93	3.82	0.08	1	0.07	58	96(R)
IRED-97	0.00	0.00	1	0.00	N.D.	N.D.
IRED-98	0.00	0.00	1	0.00	N.D.	N.D.
IRED-99	0.56	0.00	1	0.00	8	99(R)
IRED-100	0.00	0.00	1	0.00	N.D.	N.D.
IRED-101	4.42	0.10	1	0.09	68	96(R)
IRED-102	1.99	0.08	1	0.07	31	93(R)
IRED-104	2.10	0.10	1	0.09	33	91(R)
IRED-105	1.60	0.00	1	0.00	23	99(R)
IRED-107	0.00	0.00	1	0.00	N.D.	N.D.
IRED-108	3.17	0.10	1	0.09	49	94(R)
IRED-109	0.00	0.00	1	0.00	N.D.	N.D.
IRED-110	2.78	0.06	1	0.06	42	96(R)
IRED-111	4.95	0.10	1	0.09	76	96(R)
IRED-112	0.00	0.00	1	0.00	N.D.	N.D.
IRED-113	4.90	0.13	1	0.12	76	95(R)
IRED-114	4.31	0.09	1	0.08	66	96(R)
IRED-115	0.00	0.00	1	0.00	N.D.	N.D.
IRED-116	0.00	0.00	1	0.00	N.D.	N.D.
IRED-117	6.50	0.18	1	0.17	99	95(R)
IRED-118	0.00	0.00	1	0.00	N.D.	N.D.

IRED-119	3.74	0.00	1	0.00	56	99(R)
IRED-120	4.80	0.10	1	0.09	74	96(R)
IRED-121	0.00	0.00	1	0.00	N.D.	N.D.
IRED-124	0.00	0.00	1	0.00	N.D.	N.D.
IRED-125	0.00	0.00	1	0.00	N.D.	N.D.
IRED-126	0.00	0.00	1	0.00	N.D.	N.D.
IRED-127	0.00	0.00	1	0.00	N.D.	N.D.
IRED-129	0.00	0.00	1	0.00	N.D.	N.D.
IRED-130	7.37	0.00	1	0.00	99	99(R)
IRED-131	0.00	0.00	1	0.00	N.D.	N.D.
IRED-132	5.43	0.13	1	0.12	84	96(R)
IRED-133	3.98	0.11	1	0.10	61	95(R)
IRED-134	4.88	0.09	1	0.08	75	97(R)
IRED-135	5.78	0.06	1	0.06	88	98(R)
IRED-136	5.84	0.14	1	0.13	90	96(R)
IRED-138	0.00	0.00	1	0.00	N.D.	N.D.
IRED-139	5.09	0.12	1	0.11	78	96(R)
IRED-140	4.29	0.10	1	0.09	66	96(R)
IRED-142	0.00	0.00	1	0.00	N.D.	N.D.
IRED-143	3.35	0.05	1	0.05	51	97(R)
IRED-144	2.92	0.00	1	0.00	44	99(R)
IRED-145	0.00	0.00	1	0.00	N.D.	N.D.
IRED-146	1.96	0.00	1	0.00	29	99(R)
IRED-147	0.00	0.00	1	0.00	N.D.	N.D.
IRED-148	2.86	0.05	1	0.05	43	97(R)
IRED-149	2.01	0.00	1	0.00	30	99(R)
IRED-150	0.00	0.00	1	0.00	N.D.	N.D.
IRED-151	0.00	0.00	1	0.00	N.D.	N.D.
IRED-152	0.00	0.00	1	0.00	N.D.	N.D.
IRED-153	0.00	0.00	1	0.00	N.D.	N.D.
IRED-154	5.76	0.00	1	0.00	87	99(R)
IRED-155	4.79	0.11	1	0.10	74	96(R)
IRED-156	2.47	0.04	1	0.04	37	97(R)
IRED-157	4.16	0.07	1	0.07	63	97(R)
IRED-158	0.00	0.00	1	0.00	N.D.	N.D.
IRED-159	0.99	0.00	1	0.00	14	99(R)
IRED-160	0.00	0.00	1	0.00	N.D.	N.D.
IRED-161	1.79	0.00	1	0.00	26	99(R)
IRED-162	4.26	0.11	1	0.10	66	95(R)
IRED-163	4.33	0.08	1	0.07	66	97(R)
IRED-164	6.17	0.42	1	0.39	99	88(R)
IRED-165	1.95	0.10	1	0.09	30	91(R)
IRED-168	3.48	0.24	1	0.22	56	88(R)

IRED-169	6.18	0.20	1	0.19	96	94(R)
IRED-170	0.00	0.00	1	0.00	N.D.	N.D.
IRED-171	0.00	0.00	1	0.00	N.D.	N.D.
IRED-172	2.30	0.09	1	0.08	35	93(R)
IRED-174	1.47	0.09	1	0.08	23	89(R)
IRED-176	1.38	0.00	1	0.00	20	99(R)
IRED-179	0.00	0.00	1	0.00	N.D.	N.D.
IRED-180	2.26	0.09	1	0.08	35	93(R)
IRED-181	6.72	0.31	1	0.29	99	92(R)
IRED-182	1.17	0.81	1	0.76	29	21(R)
IRED-183	0.00	0.00	1	0.00	N.D.	N.D.
IRED-184	2.55	0.11	1	0.10	40	92(R)
IRED-185	6.38	0.15	1	0.14	99	96(R)
IRED-186	1.05	1.47	1	1.37	37	13(S)
IRED-188	6.40	0.20	1	0.19	99	94(R)
IRED-189	0.28	0.00	1	0.00	3	99(R)
IRED-191	0.09	1.88	1	1.76	29	90(S)
IRED-192	0.06	0.64	1	0.60	10	82(S)
IRED-194	2.31	0.18	1	0.17	37	86(R)
IRED-195	0.00	0.00	1	0.00	N.D.	N.D.

Reaction conditions: 300 μL reaction volume, substrate **1** (10 mM), $NADP^+$ (0.5 mM), BmGDH (1 mg/mL), D-glucose (30 mM), potassium phosphate buffer (100 mM, pH = 9.0), 800 rpm, 30 °C, 12 h. The yields and *ee* values were quantified by ^{19}F NMR analysis. N.D. indicates "not detected."

Evaluation of Enantiomeric Excess Values and Yields Assessment of Product 2a

a) Establishment of the method to determine the *ee* values of **2a** via ^{19}F NMR

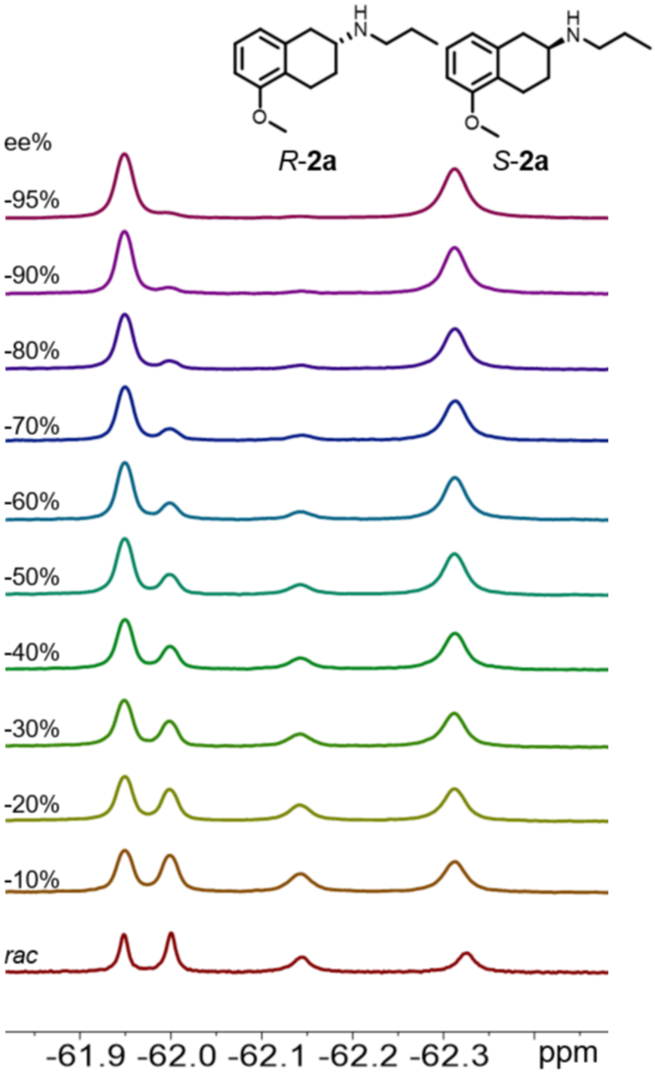

Figure S6. Establishment of the method to determine the *ee* values of **2a**. ^{1}H-decoupled ^{19}F NMR spectra of a mixture **probe-CF$_3$** (9 μmol), 2-methylpyrrolidine (ca. 5 μmol) of varying *ee* values in $CDCl_3$. ^{19}F NMR Spectra were recorded on a Bruker Avance-600 NMR spectrometer (565 MHz for the ^{19}F nucleus) using a scan number of 64.

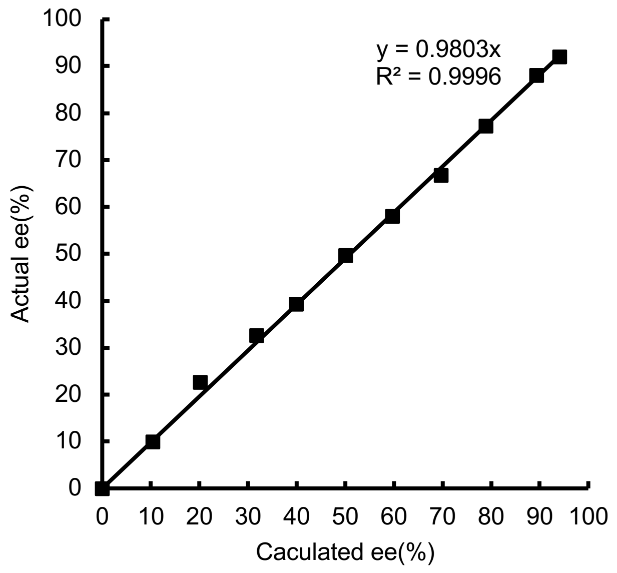

Figure S7. Linear correlation between measured *ee* and actual *ee* of product **2a**.

b) Establishment of the method to determine the yields of **2a** via ^{19}F NMR

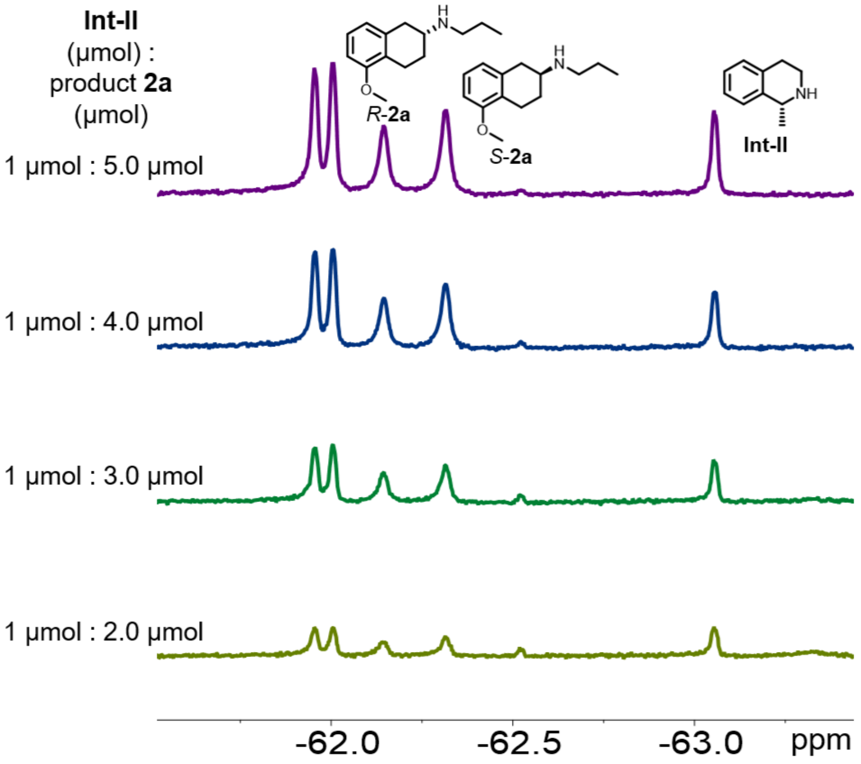

Figure S8. ^{1}H-decoupled ^{19}F NMR spectra of a mixture of **probe-CF$_3$** (ca. 9 μmol), varying amounts of product **2a** (2 to 5 μmol) and 1 μmol of internal standard (*R*)-1-methyl-1,2,3,4-tetrahydroisoquinoline (**Int-II**) in $CDCl_3$. ^{19}F NMR Spectra were recorded on a Bruker Avance-600 NMR spectrometer (565 MHz for the ^{19}F nucleus) using a scan number of 64.

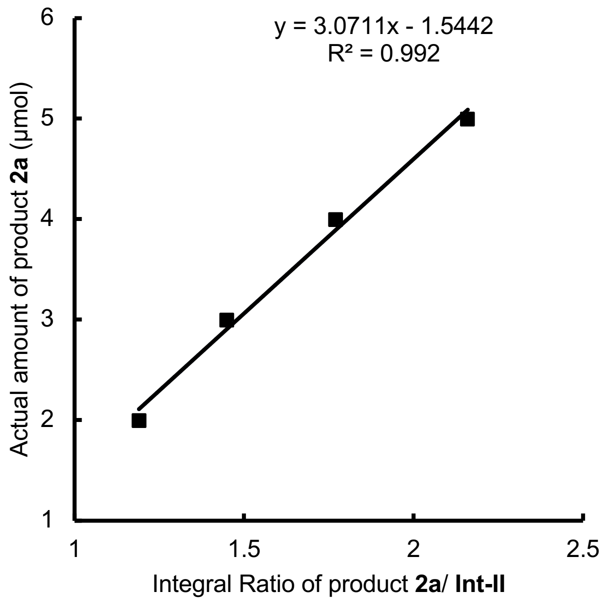

Figure S9. Linear correlation between ^{19}F NMR integral ratios of product **2a** and **Int-II** versus actual amounts of product **2a**

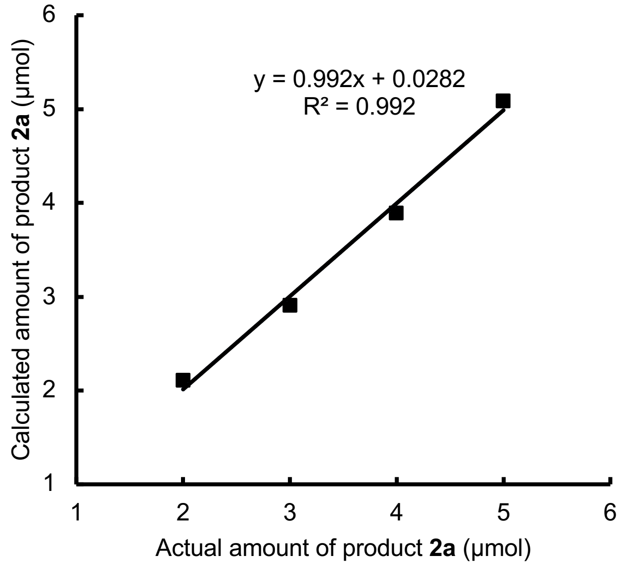

Figure S10. Linear correlation between calculated and actual amounts of product **2a**.

c) Exemplified Illustration of Enantiomeric Excess Values and Yields of **2a** via ^{19}F NMR

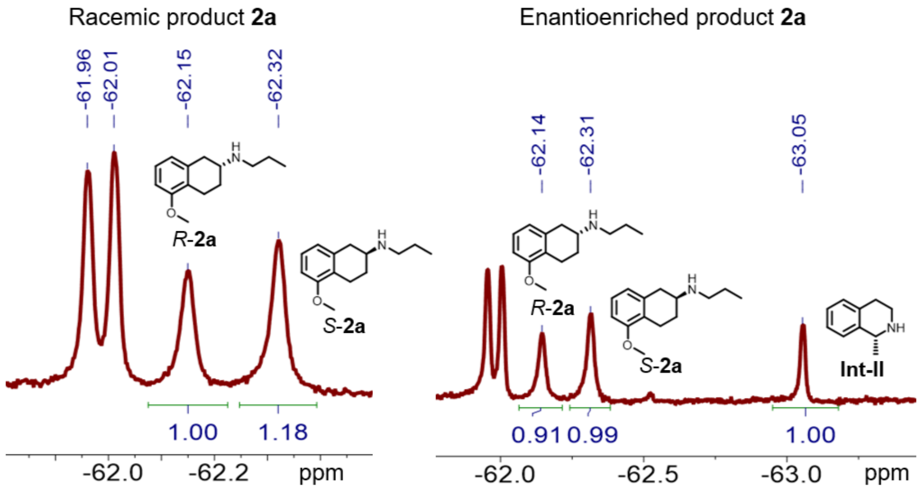

correction coefficient (**CC**)

CC = C_R/C_S = 1/1.18 = 0.847

correction integral (**I'_R**)

$I'_R = I'_R/CC$ = 0.91/0.847 = 1.07

calculated ***ee*** (%) = ($I'_R - I_S$)/($I'_R + I_S$) = **4.2%**

calculated **amount** (μmol) = 3.0711 × ($I_R + I_S$) – 1.5442 = **4.29 μmol**

calculated **yield**(%) = calculated amount/ theoretical amount ×100% = **85.8%**

Figure S11. Evaluation of enantiomeric excess values and yields of product **2a**. ^{19}F NMR Spectra were recorded on a Bruker Avance-600 NMR spectrometer (565 MHz for the ^{19}F nucleus) using a scan number of 64.

Screening of Yields and Enantiomeric Excess values of Product 2a in Directed Evolution via ^{19}F NMR Analysis

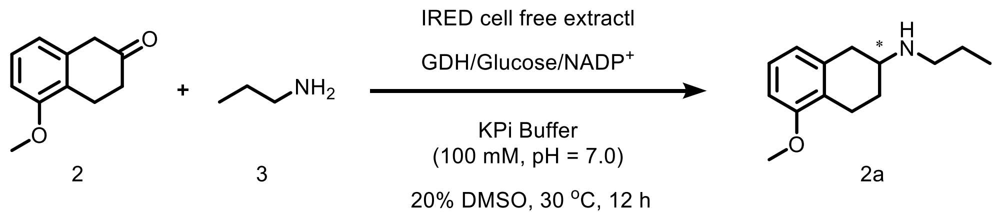

Table S2. Yields and *ee* values of the screened biosynthetic reactions to produce product **2a** as determined by ^{19}F NMR

Enzyme	Integral area of (*R*)-**2a**	Integral area of (*S*)-**2a**	Integral area of **Int-II**	Yield (%)	*ee* (%)
IRED-195	0.66	1.81	1	>99	40(S)
IRED-99	1.22	0.3	1	62	66(R)
IRED-105	0.84	0.3	1	39	54(R)

Reaction conditions: 500 µL reaction volume, cell-free extract 200 µL, substrate **2** (10 mM), *n*-propylamine (1 M), $NADP^+$ (0.5 mM), BmGDH (1 mg/mL), D-glucose (30 mM), potassium phosphate buffer (100 mM, pH = 7.0), DMSO (20%, v/v), 800 rpm, 30 °C, 12 h. The yields and *ee* values were quantified by ^{19}F NMR analysis.

Table S3. Yields and *ee* values determined in the direct evolution of IRED-195

Mutant	Integral area of (*R*)-**2a**	Integral area of (*S*)-**2a**	Integral area of **Int-II**	Yield (%)	*ee* (%)
T101S	0.33	1.41	1	76	57(S)
I129C	0.71	1	1	74	9(S)
I129F	0.79	1.34	1	>99	18(S)
I129L	0.78	1.27	1	95	16(S)
I129M	0.8	1.33	1	>99	17(S)
I129P	0.41	1.46	1	84	50(S)
I129T	1.13	1.71	1	>99	12(S)
I129W	1.84	0.97	1	>99	38(R)
I129Y	1.08	1.1	1	>99	7(R)
M183A	0.92	1.32	1	>99	10(S)
M183C	0.72	1.54	1	>99	29(S)
M183D	0.64	1.16	1	80	21(S)
M183F	1.3	0.87	1	>99	28(R)
M183G	0.63	1.38	1	93	30(S)
M183H	1.31	0.85	1	>99	29(R)
M183I	0.61	1.58	1	>99	37(S)
M183L	0.97	0.93	1	86	10(R)
M183N	0.73	1.37	1	98	23(S)
M183S	0.73	1.24	1	90	18(S)

M183T	0.69	1.41	1	98	27(S)
M183V	0.68	1.22	1	86	21(S)
M183W	1.35	1	1	>99	23(R)
M183Y	1.3	0.98	1	>99	22(R)
Y184A	0.92	1.21	1	>99	5(S)
Y184C	1.09	1.08	1	>99	9(R)
Y184E	0.73	2.27	1	>99	45(S)
Y184F	0.82	1.22	1	94	12(S)
Y184G	0.96	1	1	90	6(R)
Y184L	0.94	0.96	1	86	7(R)
Y184M	0.78	1.05	1	82	7(S)
Y184N	0.63	0.74	1	53	rac
Y184Q	1	1.3	1	>99	5(S)
Y184S	0.83	1.12	1	89	7(S)
Y184T	1.14	1.82	1	>99	15(S)
Y184V	0.83	1.16	1	91	8(S)
W214A	0.57	0.79	1	53	8(S)
W214C	0.8	1.22	1	93	13(S)
W214F	0.9	1.27	1	>99	9(S)
W214I	1.1	1.09	1	>99	8(R)
W214L	5.25	5.35	1	>99	7(R)
W214M	0.92	1.34	1	>99	10(S)
W214S	2.04	4.07	1	>99	26(S)
W214T	0.7	0.89	1	67	4(S)
W214V	0.85	0.92	1	78	4(R)
G248A	0.48	2.63	1	>99	65(S)
G248C	0.29	1.95	1	>99	70(S)
G248E	0.14	1.21	1	52	76(S)
G248F	0.24	1.33	1	66	65(S)
G248H	0.35	2.38	1	>99	70(S)
G248I	0.2	1.43	1	69	72(S)
G248M	0.24	1.72	1	90	72(S)
G248N	0.19	1.33	1	62	71(S)
G248Q	0.33	2.41	1	>99	72(S)
G248S	0.33	2.33	1	>99	71(S)
G248T	0.09	2.02	1	99	90(S)
G248V	0.24	1.49	1	75	68(S)
G248W	0.21	1.26	1	60	67(S)
G248Y	0.07	1.6	1	72	90(S)
F249A	0.53	2.22	1	>99	56(S)
F249C	0.54	2.54	1	>99	60(S)
F249E	0.26	1.03	1	48	54(S)
F249G	0.64	1.59	1	>99	36(S)

F249I	0.62	2.32	1	>99	52(S)
F249L	0.62	1.79	1	>99	42(S)
F249M	0.67	1.71	1	>99	37(S)
F249N	0.45	2.62	1	>99	66(S)
F249Q	0.53	1.85	1	>99	49(S)
F249S	0.73	2.53	1	>99	49(S)
F249T	0.61	2.34	1	>99	53(S)
F249V	0.57	2.51	1	>99	58(S)
F249W	0.52	1.3	1	81	36(S)
F249Y	0.73	2.04	1	>99	40(S)

Reaction conditions: 500 μL reaction volume, cell-free extract 200 μL, substrate **3** (10 mM), *n*-propylamine (1 M), $NADP^+$ (0.5 mM), BmGDH (1 mg/mL), D-glucose (30 mM), potassium phosphate buffer (100 mM, pH = 7.0), DMSO (20%, v/v), 800 rpm, 30 °C, 12 h. The yields and *ee* values were quantified by ^{19}F NMR analysis.

Evaluation of Enantiomeric Excess Values and Yields Assessment of Product 4a

a) Establishment of the method for the determination of *ee* of **4a** via ^{19}F NMR

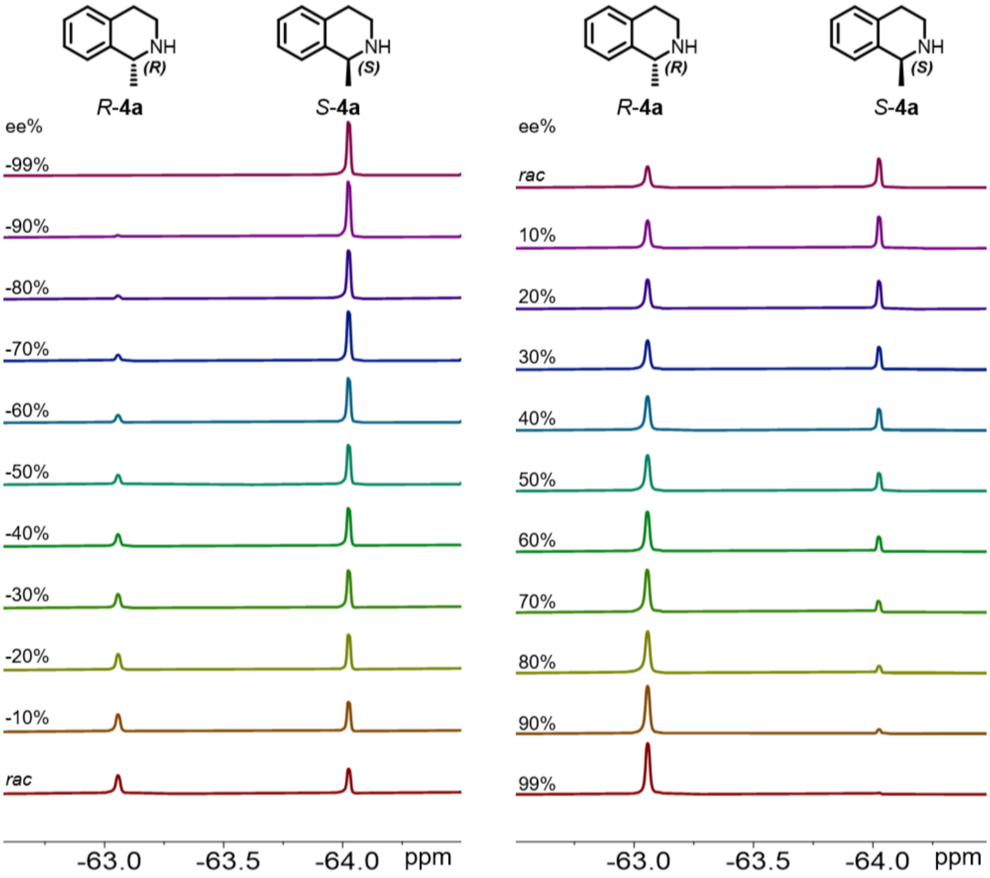

Figure S12. Establishment of the method for the determination of *ee* of **4a**. ^{1}H-decoupled ^{19}F NMR spectra of mixtures of **probe-CF$_3$** (9 μmol), 1-methyl-1,2,3,4-tetrahydroisoquinoline (ca. 6 μmol) of varying *ee* values in $CDCl_3$. ^{19}F NMR Spectra were recorded on a Bruker Avance-600 NMR spectrometer (565 MHz for the ^{19}F nucleus) using a scan number of 16.

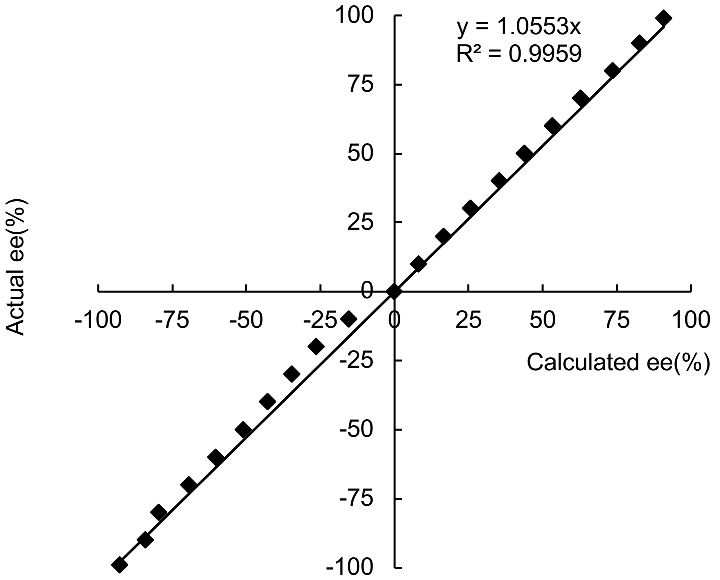

Figure S13. Linear correlation between measured and actual enantiomeric excess (*ee*) of product **4a**.

b) Establishment of the method for the determination of the yields of **4a** via ^{19}F NMR

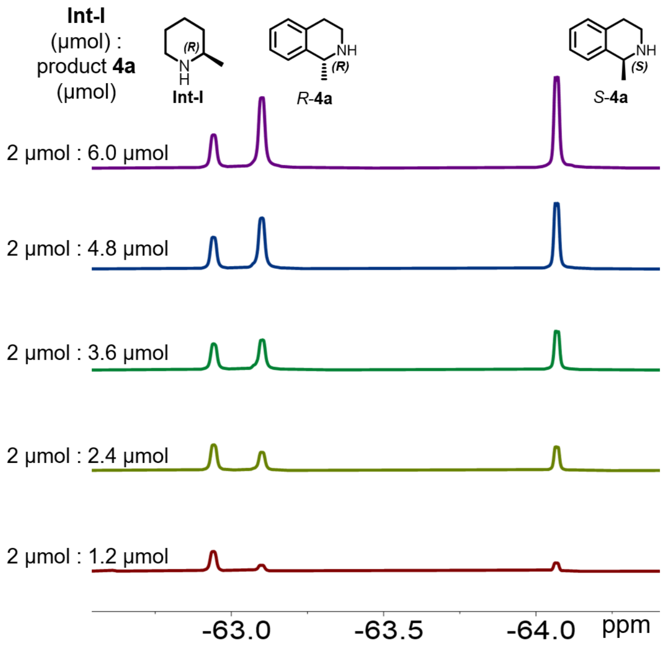

Figure S14. Establishment of the method for the determination of the yields of **4a**. ^{1}H-decoupled ^{19}F NMR spectra of mixtures of **probe-CF$_3$** (ca. 9 μmol), varying amounts of product **4a** (ca. 0 to 6 μmol) and 2 μmol of internal standard (*R*)-2-methylpiperidine (**Int-I**) in $CDCl_3$. ^{19}F NMR Spectra were recorded on a Bruker Avance-600 NMR spectrometer (565 MHz for the ^{19}F nucleus) using a scan number of 16.

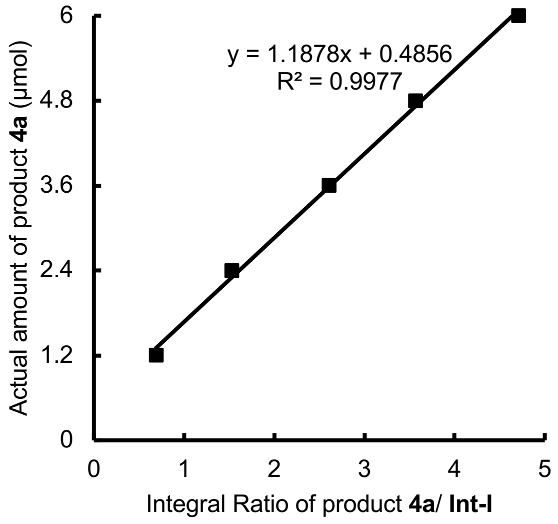

Figure S15. Linear correlation between ^{19}F NMR integral ratios of product **4a** and **Int-I** versus actual concentration of product **4a**.

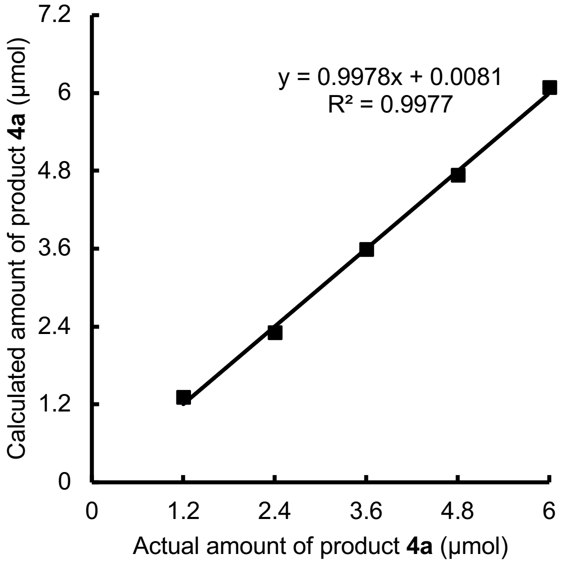

Figure S16. Linear correlation between calculated and actual amounts of product **4a**.

c) Exemplified Illustration of Enantiomeric Excess Values and Yields of **4a** via ^{19}F NMR

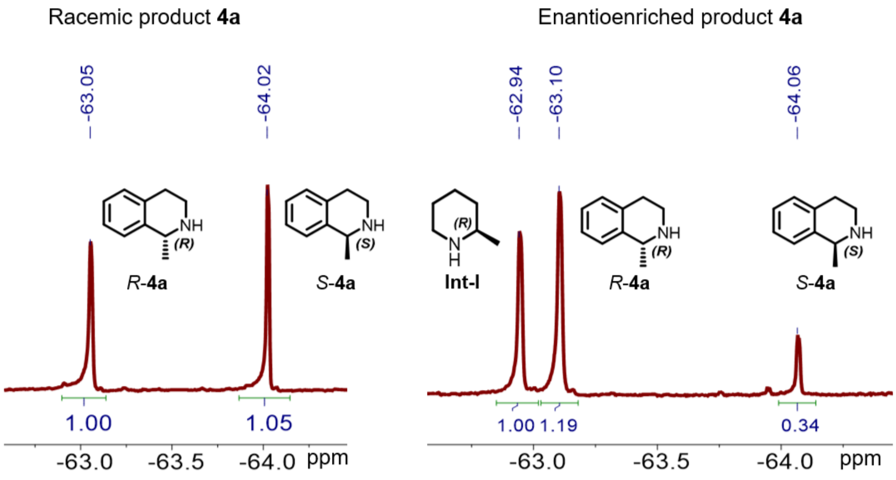

correction coefficient (**CC**)

CC = C_R/C_S = 1/1.05 = 0.952

correction integral (I'_R)

I'_R = I_R/CC = 1.19/0.952 = 1.25

calculated ***ee*** (%) = **(I'_R-I_S)/(I'_R+I_S)** = **57.2**%

calculated **amount** (μmol) = 1.1878 × (**I_R + I_S**) + 0.4856 = **3.09 μmol**

calculated **yield**(%) = calculated amount/ theoretical amount ×100% = **51.5**%

Figure S17. Evaluation of enantiomeric excess values and yields of product **4a** via ^{19}F NMR. ^{19}F NMR Spectra were recorded on a Bruker Avance-600 NMR spectrometer (565 MHz for the ^{19}F nucleus) using a scan number of 16.

Screening of Yields and Enantiomeric Excess Values of Product 4a via ^{19}F NMR Analysis

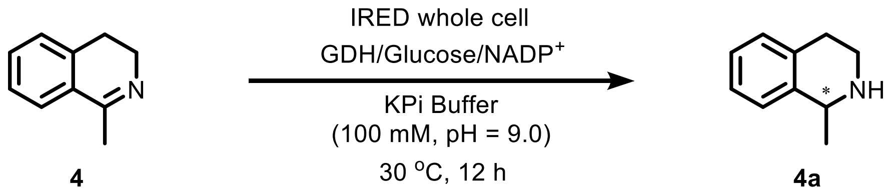

Table S4. Yields and *ee* values of **4a** produced by the screened biocatalytic reactions as determined by ^{19}F NMR

Enzyme	Intergral area of (R)-4a	Intergral area of (S)-**4a**	Integral area of **Int-I**	Corrected integral area of (S)-**4a**	Yield (%)	*ee* (%)
IRED-2	0.00	0.00	1	0.00	N.D.	N.D.
IRED-3	0.00	0.00	1	0.00	N.D.	N.D.
IRED-5	0.00	0.00	1	0.00	N.D.	N.D.
IRED-6	0.61	0.00	1	0.00	20	99(R)
IRED-8	0.81	0.00	1	0.00	24	99(R)
IRED-12	0.00	0.00	1	0.00	N.D.	N.D.
IRED-13	0.00	0.00	1	0.00	N.D.	N.D.
IRED-17	1.52	0.06	1	0.06	39	92(R)
IRED-18	1.42	0.14	1	0.14	39	82(R)
IRED-19	0.72	0.10	1	0.10	24	77(R)
IRED-20	0.95	0.00	1	0.00	27	99(R)
IRED-22	0.00	0.00	1	0.00	N.D.	N.D.
IRED-23	0.00	0.00	1	0.00	N.D.	N.D.
IRED-24	0.00	0.00	1	0.00	N.D.	N.D.
IRED-25	0.00	0.00	1	0.00	N.D.	N.D.
IRED-27	0.00	0.00	1	0.00	N.D.	N.D.
IRED-28	0.36	0.00	1	0.00	15	99(R)
IRED-31	1.36	0.15	1	0.15	38	80(R)
IRED-32	0.00	0.00	1	0.00	N.D.	N.D.
IRED-33	0	0.14	1	0.14	11	99(S)
IRED-34	0.84	0.13	1	0.13	27	74(R)
IRED-36	1.46	0.05	1	0.05	38	93(R)
IRED-37	1.12	0.00	1	0.00	30	99(R)
IRED-42	0	0.45	1	0.45	17	99(S)
IRED-44	0	0.07	1	0.07	10	99(S)
IRED-45	0	0.13	1	0.13	11	99(S)
IRED-47	1.41	0.00	1	0.00	36	99(R)
IRED-48	0.00	0.00	1	0.00	N.D.	N.D.
IRED-49	0.38	0.06	1	0.06	17	71(R)

IRED-50	1.44	0.00	1	0.00	37	99(R)
IRED-51	1.5	0.00	1	0.00	38	99(R)
IRED-52	0.18	0.00	1	0.00	12	99(R)
IRED-54	0.1	0.00	1	0.00	10	99(R)
IRED-55	1.41	0.00	1	0.00	36	99(R)
IRED-56	1.02	0.05	1	0.05	29	90(R)
IRED-59	1	0.12	1	0.12	30	79(R)
IRED-60	0.00	0.00	1	0.00	N.D.	N.D.
IRED-61	0.00	0.00	1	0.00	N.D.	N.D.
IRED-64	0.35	0.00	1	0.00	15	99(R)
IRED-66	0.56	0.00	1	0.00	19	99(R)
IRED-67	1.18	0.00	1	0.00	31	99(R)
IRED-68	0.17	0.00	1	0.00	11	99(R)
IRED-69	0.28	0.00	1	0.00	14	99(R)
IRED-70	0.00	0.00	1	0.00	N.D.	N.D.
IRED-72	0.25	0.11	1	0.11	15	40(R)
IRED-73	0.69	0.00	1	0.00	22	99(R)
IRED-74	0.51	0.00	1	0.00	18	99(R)
IRED-76	0.00	0.00	1	0.00	N.D.	N.D.
IRED-81	0.34	0.00	1	0.00	15	99(R)
IRED-83	0.28	0.00	1	0.00	14	99(R)
IRED-84	0.31	0.00	1	0.00	14	99(R)
IRED-93	0.6	0.00	1	0.00	20	99(R)
IRED-97	0.43	0.00	1	0.00	17	99(R)
IRED-98	0.59	0.00	1	0.00	20	99(R)
IRED-99	0.67	0.10	1	0.10	23	75(R)
IRED-100	0.75	0.11	1	0.11	25	75(R)
IRED-101	0.67	0.10	1	0.10	23	75(R)
IRED-102	0.34	0.07	1	0.07	16	64(R)
IRED-104	0.71	0.00	1	0.00	22	99(R)
IRED-105	0.64	0.07	1	0.07	22	79(R)
IRED-107	0.46	0.00	1	0.00	17	99(R)
IRED-108	1.34	0.32	1	0.32	41	62(R)
IRED-109	0.28	0.00	1	0.00	14	99(R)
IRED-110	1.36	0.29	1	0.29	41	65(R)
IRED-111	1.33	0.13	1	0.13	37	83(R)
IRED-112	0.34	0.05	1	0.05	16	73(R)
IRED-113	1.5	0.06	1	0.06	39	92(R)
IRED-114	0.39	0.00	1	0.00	16	99(R)
IRED-115	1.45	0.06	1	0.06	38	92(R)
IRED-116	0.00	0.00	1	0.00	N.D.	N.D.
IRED-117	1.3	0.12	1	0.12	36	84(R)

IRED-118	0.00	0.00	1	0.00	N.D.	N.D.
IRED-119	0.68	0.00	1	0.00	22	99(R)
IRED-120	1.28	0.21	1	0.21	38	72(R)
IRED-121	1.36	0.10	1	0.10	37	87(R)
IRED-124	0.85	0.00	1	0.00	25	99(R)
IRED-125	0.56	0.10	1	0.10	21	71(R)
IRED-126	0.00	0.00	1	0.00	N.D.	N.D.
IRED-127	0.62	0.00	1	0.00	20	99(R)
IRED-129	0.2	0.00	1	0.00	12	99(R)
IRED-130	1.34	0.15	1	0.15	38	80(R)
IRED-131	0.97	0.12	1	0.12	30	79(R)
IRED-132	1.33	0.12	1	0.12	37	84(R)
IRED-133	0.67	0.00	1	0.00	21	99(R)
IRED-134	1.45	0.08	1	0.08	38	89(R)
IRED-135	1.29	0.00	1	0.00	34	99(R)
IRED-136	1.39	0.08	1	0.08	37	88(R)
IRED-138	1.42	0.06	1	0.06	37	91(R)
IRED-139	1.22	0.07	1	0.07	34	89(R)
IRED-140	0.32	0.00	1	0.00	14	99(R)
IRED-142	0.92	0.00	1	0.00	26	99(R)
IRED-143	1.37	0.00	1	0.00	35	99(R)
IRED-144	0.23	0.00	1	0.00	13	99(R)
IRED-145	0.32	0.00	1	0.00	14	99(R)
IRED-146	1.34	0.00	1	0.00	35	99(R)
IRED-147	0.00	0.00	1	0.00	N.D.	N.D.
IRED-148	1.4	0.10	1	0.10	38	87(R)
IRED-149	0.82	0.06	1	0.06	26	86(R)
IRED-150	0.34	0.00	1	0.00	15	99(R)
IRED-151	0.16	0.00	1	0.00	11	99(R)
IRED-152	0.34	0.00	1	0.00	15	99(R)
IRED-153	0.00	0.00	1	0.00	N.D.	N.D.
IRED-154	1.27	0.00	1	0.00	33	99(R)
IRED-155	1.39	0.08	1	0.08	37	88(R)
IRED-156	0.62	0.00	1	0.00	20	99(R)
IRED-157	1.45	0.07	1	0.07	38	90(R)
IRED-158	1.44	0.07	1	0.07	38	90(R)
IRED-159	1.39	0.00	1	0.00	36	99(R)
IRED-160	1.44	0.07	1	0.07	38	90(R)
IRED-161	1.52	0.05	1	0.05	39	93(R)
IRED-162	1.36	0.00	1	0.00	35	99(R)
IRED-163	1.41	0.00	1	0.00	36	99(R)
IRED-164	0.91	0.00	1	0.00	26	99(R)

IRED-165	0.26	0.00	1	0.00	13	99(R)
IRED-168	0.46	0.00	1	0.00	17	99(R)
IRED-169	1.48	0.18	1	0.18	41	78(R)
IRED-170	0.00	0.00	1	0.00	N.D.	N.D.
IRED-171	0.00	0.00	1	0.00	N.D.	N.D.
IRED-172	0.31	0.00	1	0.00	14	99(R)
IRED-174	0.27	0.00	1	0.00	13	99(R)
IRED-176	0.87	0.00	1	0.00	25	99(R)
IRED-179	1.48	0.00	1	0.00	37	99(R)
IRED-180	0.36	0.23	1	0.23	20	21(R)
IRED-181	0	0.24	1	0.24	13	99(S)
IRED-182	0	0.61	1	0.61	20	99(S)
IRED-184	0.00	0.00	1	0.00	N.D.	N.D.
IRED-185	1.36	0.15	1	0.15	38	80(R)
IRED-186	0.14	1.55	1	1.55	42	83(S)
IRED-188	1.24	0.15	1	0.15	36	79(R)
IRED-189	0.00	0.00	1	0.00	N.D.	N.D.
IRED-191	0	0.90	1	0.90	26	99(S)
IRED-192	0	0.21	1	0.21	12	99(S)
IRED-194	0.81	0.15	1	0.15	27	69(R)
IRED-195	0.34	0.00	1	0.00	15	99(R)

Reaction conditions: 300 µL reaction volume, substrate **4** (10 mM), $NADP^+$ (0.5 mM), BmGDH (1 mg/mL), D-glucose (30 mM), potassium phosphate buffer (100 mM, pH = 9.0), 800 rpm, 30 °C, 12 h. The yields and *ee* values were quantified by ^{19}F NMR analysis. N.D. indicates "not detected".

Sequence Alignment Stereoselective IREDs

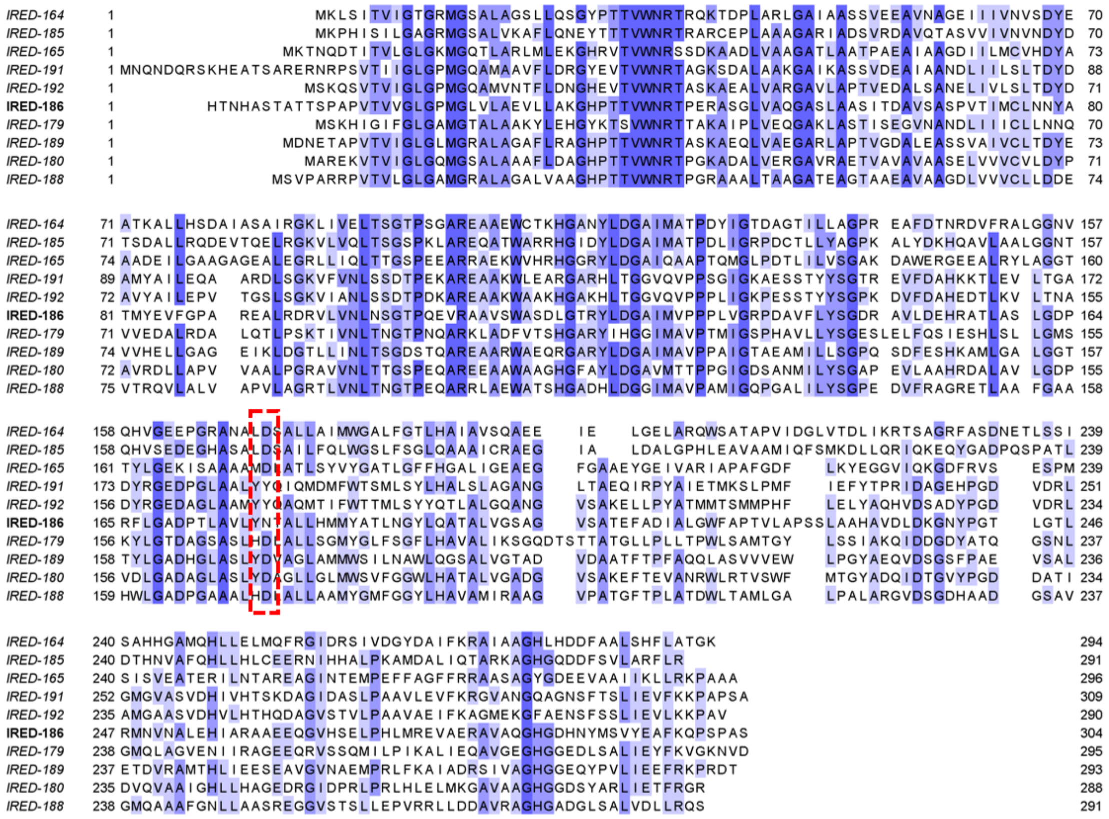

Figure S18. Sequence alignment of stereoselective imine reductases (IREDs) using Clustal Omega.

Structural Overlays and Active Site Comparisons of IREDs

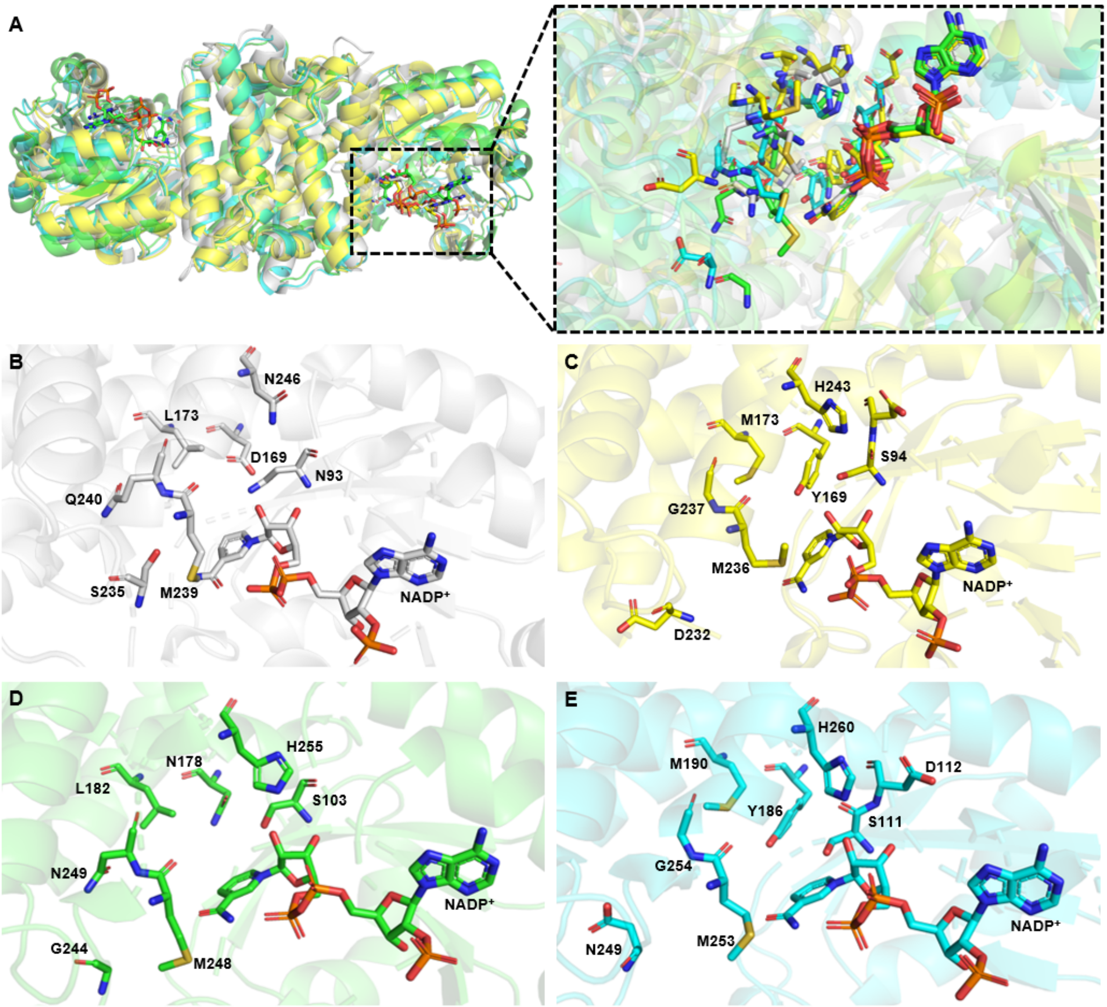

Figure S19. Structural Overlays and Active Site Comparisons of IREDs (A) Structural overlay of IRED-191 (cyan), homology modeled based on PDB ID: 4D3D using SWISS-MODEL, with known imine reductases PDB ID 5G6S (white), 7OG3 (green), and 4OQY (yellow). (B-E) Detailed views of the cofactor and surrounding residues in the active sites of PDB ID 5G6S(B), 4OQY(C), 7OG3(D), and IRED-191(E).

Chromatogram

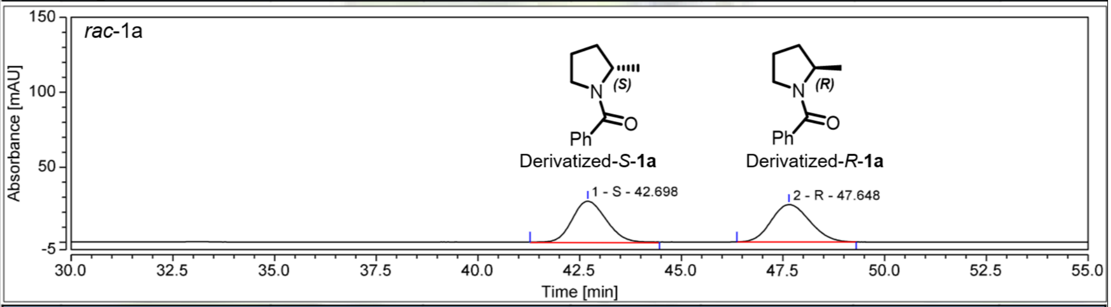

Integration Results

No.	Peak Name	Retention Time min	Area mAU*min	Height mAU	Relative Area %	Relative Height %	Amount
1	S	42.698	27.071	27.589	49.73	52.55	n.a.
2	R	47.648	27.360	24.914	50.27	47.45	n.a.
**Total:**			**54.430**	**52.503**	**100.00**	**100.00**	

Figure S20. Chiral HPLC analysis of racemic standard of derivatized-**1a**. Product **1a** was subjected to derivatization by treatment with benzoyl chloride and triethylamine. HPLC conditions: CHIRALPAK IC column with a mobile phase of *n*-hexane/ *i*-Propanol (90:10, v/v), flow rate 1.0 mL/min, 30 °C, UV detection at 254 nm. Retention time (TR) for R and S enantiomers are 47.648 min (major) and 42.698 min (minor), and peak areas of S and R enantiomers are 49.73 % and 50.27 % respectively. The *ee* value measured by HPLC is 3 %.

Chromatogram

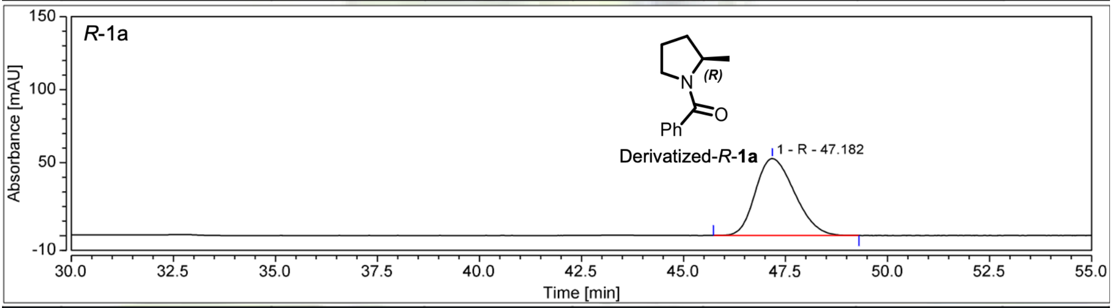

Integration Results

No.	Peak Name	Retention Time min	Area mAU*min	Height mAU	Relative Area %	Relative Height %	Amount
n.a.	S	n.a.	n.a.	n.a.	n.a.	n.a.	n.a.
1	R	47.182	58.727	52.678	100.00	100.00	n.a.
**Total:**			**58.727**	**52.678**	**100.00**	**100.00**	

Figure S21. Chiral HPLC analysis of optically pure standard of derivatized-(*R*)-**1a**. Product **1a** was subjected to derivatization by treatment with benzoyl chloride and triethylamine. HPLC conditions: CHIRALPAK IC column with a mobile phase of *n*-hexane/ *i*-Propanol (90:10, v/v), flow rate 1.0 mL/min, 30 °C, UV detection at 254 nm. Retention time (TR) for R enantiomers are 47.182 min (major), and peak areas of S and R enantiomers are 100 %. The *ee* value measured by HPLC is 100 %.

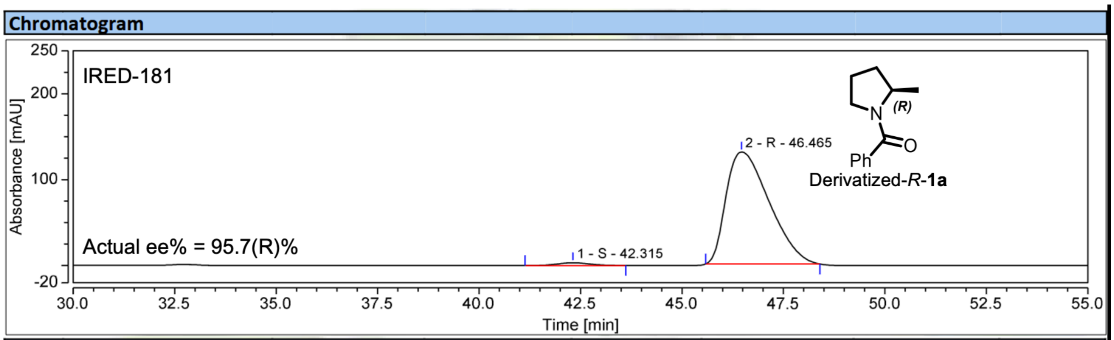

Integration Results

No.	Peak Name	Retention Time min	Area mAU*min	Height mAU	Relative Area %	Relative Height %	Amount
1	S	42.315	3.512	3.371	2.14	2.52	n.a.
2	R	46.465	160.700	130.401	97.86	97.48	n.a.
Total:			**164.212**	**133.772**	**100.00**	**100.00**	

Figure S22. Chiral HPLC analysis of IRED-181 catalytic derivatized-**1a**. Product **1a** was subjected to derivatization by treatment with benzoyl chloride and triethylamine. HPLC conditions: CHIRALPAK IC column with a mobile phase of *n*-hexane/ *i*-Propanol (90:10, v/v), flow rate 1.0 mL/min, 30 °C, UV detection at 254 nm. Retention time (TR) for R and S enantiomers are 46.465 min (major) and 42.315 min (minor), and peak areas of S and R enantiomers are 2.14 % and 97.86 % respectively. The *ee* value measured by HPLC is -95.7%.

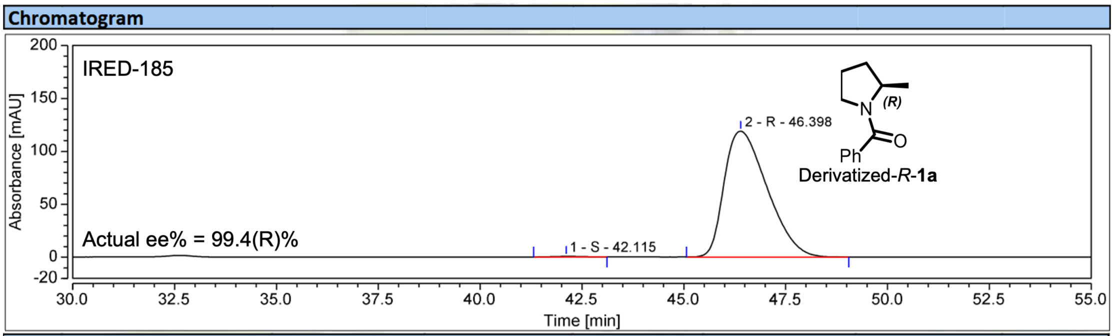

Integration Results

No.	Peak Name	Retention Time min	Area mAU*min	Height mAU	Relative Area %	Relative Height %	Amount
1	S	42.115	0.466	0.546	0.32	0.46	n.a.
2	R	46.398	145.286	118.902	99.68	99.54	n.a.
Total:			**145.752**	**119.447**	**100.00**	**100.00**	

Figure S23. Chiral HPLC analysis of IRED-185 catalytic derivatized-**1a**. Product **1a** was subjected to derivatization by treatment with benzoyl chloride and triethylamine. HPLC conditions: CHIRALPAK IC column with a mobile phase of *n*-hexane/ *i*-Propanol (90:10, v/v), flow rate 1.0 mL/min, 30 °C, UV detection at 254 nm. Retention time (TR) for R and S enantiomers are 46.398 min (major) and 42.115 min (minor), and peak areas of S and R enantiomers are 0.32 % and 99.68 % respectively. The *ee* value measured by HPLC is 99.3%.

Chromatogram

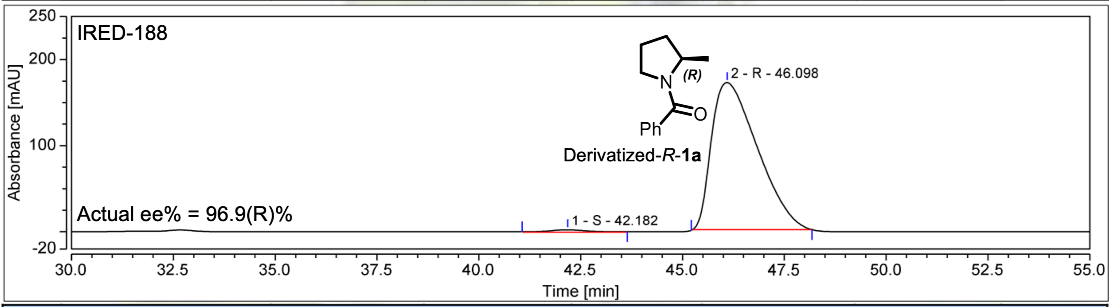

Integration Results

No.	Peak Name	Retention Time min	Area mAU*min	Height mAU	Relative Area %	Relative Height %	Amount
1	S	42.182	3.557	2.828	1.57	1.63	n.a.
2	R	46.098	223.212	170.925	98.43	98.37	n.a.
**Total:**			**226.769**	**173.753**	**100.00**	**100.00**	

Figure S24. Chiral HPLC analysis of IRED-188 catalytic derivatized-**1a**. Product **1a** was subjected to derivatization by treatment with benzoyl chloride and triethylamine. HPLC conditions: CHIRALPAK IC column with a mobile phase of *n*-hexane/ *i*-Propanol (90:10, v/v), flow rate 1.0 mL/min, 30 °C, UV detection at 254 nm. Retention time (TR) for R and S enantiomers are 46.098 min (major) and 42.182 min (minor), and peak areas of S and R enantiomers are 1.57 % and 98.43% respectively. The *ee* value measured by HPLC is -96.9 %.

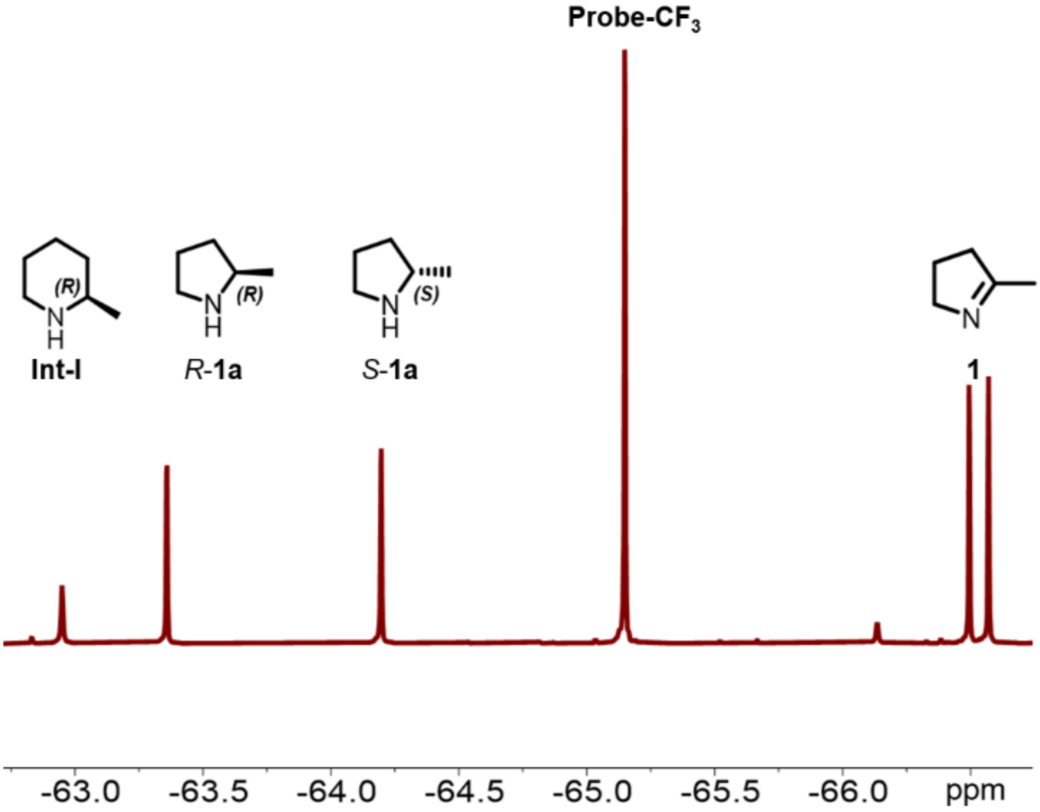

Figure S25. ^{19}F NMR spectrum of a mixture of substrate, products, internal standard and **Probe-CF$_3$**. ^{19}F NMR spectrum was recorded on a Bruker Avance-600 NMR spectrometer (565 MHz for the ^{19}F nucleus) using a scan number of 16.

A Comparison Between Enantiomeric Excess Values 1a Determined by ^{19}F NMR and Chiral HPLC Analysis

Table S5. *ee* values of product **1a** determined by ^{19}F NMR or chiral HPLC analysis

Enzyme	*ee* (%) determined by NMR	*ee* (%) determined by HPLC
IRED-181	96.3(R)	95.7(R)
IRED-185	99(R)	99.3(R)
IRED-188	96.3(R)	96.9(R)

Exemplified High Throughput Screening of Enantiocomposition of 1a via ^{19}F NMR

For NMR analysis with shimming and locking, a typical ^{19}F NMR analysis requires approximately 4 mins. For the high throughput analysis without shimming and locking, each sample requires 87 seconds (24 samples in 35 mins), which provides the same accuracy compared to the analysis with locking and shimming. This benefits from the free of background signal of ^{19}F NMR.

Table S6. Yield and *ee* result of product **1a** determined by ^{19}F NMR under different conditions

	^{19}F NMR with shimming and locking (4 minutes per sample)		^{19}F NMR without shimming and locking (87 seconds per sample)	
Enzyme	Yield(%)	*ee*(%)	Yield(%)	*ee*(%)
IRED-2	N.D.	N.D.	N.D.	N.D.
IRED-3	26.7	99(R)	26.1	99(R)
IRED-5	N.D.	N.D.	N.D.	N.D.
IRED-8	52.6	97(R)	50.9	95(R)
IRED-12	N.D.	N.D.	N.D.	N.D.
IRED-13	N.D.	N.D.	N.D.	N.D.
IRED-17	N.D.	N.D.	N.D.	N.D.
IRED-18	48.5	93(R)	47.9	92(R)
IRED-19	29.4	99(R)	32.1	99(R)
IRED-22	N.D.	N.D.	N.D.	N.D.
IRED-23	N.D.	N.D.	N.D.	N.D.
IRED-24	N.D.	N.D.	N.D.	N.D.
IRED-27	N.D.	N.D.	N.D.	N.D.
IRED-28	50.8	96(R)	49.9	96(R)
IRED-31	42.6	93(R)	41.8	93(R)
IRED-32	N.D.	N.D.	N.D.	N.D.
IRED-33	N.D.	N.D.	N.D.	N.D.
IRED-34	46.7	94(R)	48.4	94(R)
IRED-36	40.0	99(R)	38.3	99(R)
IRED-37	N.D.	N.D.	N.D.	N.D.
IRED-42	N.D.	N.D.	N.D.	N.D.
IRED-51	38.0	95(R)	36.8	97(R)
IRED-60	21.4	88(R)	23.7	90(R)
IRED-72	39.8	99(R)	40.5	99(R)

Reaction conditions: 300 µL reaction volume, substrate **1** (10 mM), $NADP^+$ (0.5 mM), BmGDH (1 mg/mL), *D*-glucose (30 mM), potassium phosphate buffer (100 mM, pH = 9.0), 800 rpm, 30 °C, 12 h. The yields and *ee* values were quantified by ^{19}F NMR analysis using a scan number of 16. N.D. indicates "not detected".

Validation of the ^{19}F NMR Platform in Whole-Cell-Based Biocatalytic Systems

Table S7. Yield and *ee* result of product **1a** determined by ^{19}F NMR in whole cell and cell lysate

Enzyme system	Intergral area of (R)-**1a**	Intergral area of (S)-**1a**	Integral area of **Int-I**	Corrected integral area of (S)-**1a**	Yield(%)	ee(%)
Whole-cell	0.33	6.2	1	5.79	99	89.2(S)
cell lysate	0.37	6.61	1	6.25	99	88.7(S)

Reaction conditions: 300 μL reaction volume, substrate **1** (10 mM), $NADP^+$ (0.5 mM), BmGDH (1 mg/mL), *D*-glucose (30 mM), 100 mg/mL *E. coli* whole-cells or 1 mg/ml cell lysate expressing IRED-191, potassium phosphate buffer (100 mM, pH = 9.0), 800 rpm, 30 °C, 12 h. The yields and *ee* values were quantified by ^{19}F NMR analysis using a scan number of 16.

Performance of WT and Mutants of IRED-195

Table S8. Yield and *ee* result of product **2a** determined by ^{19}F NMR

Mutant	Substrate loading(mM)	Yield(%)	ee(%)
IRED-195(WT)	10	99	40(S)
IRED-195-R1(T101S-218M)	50	36	99(S)
IRED-195-R2(T101S-I129F-A218M-F249N-L252I-M273I)	50	91	99(S)

Reaction conditions: 500 μL reaction volume, substrate **2** (10 mM or 50 mM), amine **3** (1M for 10 mM substrate **2** and 0.5 M for 50mM substrate **2**), $NADP^+$ (1 mM for 10 mM substrate **2** and 2.5mM for 50mM substrate **2**), BmGDH (1 mg/mL), *D*-glucose (30 mM for 10mM substrate **2** and 150 mM for 50 mM substrate **2**), 1 mg/ml cell lysate, potassium phosphate buffer (100 mM, pH = 7.0), DMSO (20%, v/v), 800 rpm, 30 °C, 12 h. The yields and *ee* values were quantified by ^{19}F NMR analysis using a scan number of 64.

Table S9. Yield and *ee* result of product **2a** in preparative scale reaction determined by ^{19}F NMR

Mutant	Substrate loading(mM)	Yield(%)	ee(%)
IRED-195-R2(T101S-I129F-A218M-F249N-L252I-M273I)	50	72	99(S)

Reaction conditions: 50 mL reaction volume, substrate **2** (50 mM), amine **3** (0.5 M), $NADP^+$ (2.5 mM), BmGDH (1 mg/mL), *D*-glucose (150 mM), 1 mg/mL lyophilized lysate, potassium phosphate buffer (100 mM, pH = 7.0), DMSO (20%, v/v), 220 rpm, 30 °C, 24 h.

Primers Used for Site-Saturation Mutagenesis of 9 Sites

Table S10. Primers used for site-saturation mutagenesis of 9 sites

Primer	Sequence (5' to 3')
101A-F	CTGGTTAACCTGACCGCAGGTACTCCGAAACAGGCTC
101C-F	CTGGTTAACCTGACCTGCGGTACTCCGAAACAGGCTC
101D-F	CTGGTTAACCTGACCGATGGTACTCCGAAACAGGCTC
101E-F	CTGGTTAACCTGACCGAAGGTACTCCGAAACAGGCTC
101F-F	CTGGTTAACCTGACCTTTGGTACTCCGAAACAGGCTC
101G-F	CTGGTTAACCTGACCGGTGGTACTCCGAAACAGGCTC
101H-F	CTGGTTAACCTGACCCATGGTACTCCGAAACAGGCTC
101I-F	CTGGTTAACCTGACCATTGGTACTCCGAAACAGGCTC
101K-F	CTGGTTAACCTGACCAAAGGTACTCCGAAACAGGCTC
101L-F	CTGGTTAACCTGACCCTGGGTACTCCGAAACAGGCTC
101M-F	CTGGTTAACCTGACCATGGGTACTCCGAAACAGGCTC
101N-F	CTGGTTAACCTGACCAACGGTACTCCGAAACAGGCTC
101P-F	CTGGTTAACCTGACCCCGGGTACTCCGAAACAGGCTC
101Q-F	CTGGTTAACCTGACCCAGGGTACTCCGAAACAGGCTC
101R-F	CTGGTTAACCTGACCCGTGGTACTCCGAAACAGGCTC
101S-F	CTGGTTAACCTGACCAGCGGTACTCCGAAACAGGCTC
101V-F	CTGGTTAACCTGACCGTGGGTACTCCGAAACAGGCTC
101W-F	CTGGTTAACCTGACCTGGGGTACTCCGAAACAGGCTC
101Y-F	CTGGTTAACCTGACCTATGGTACTCCGAAACAGGCTC
101-R	GGTCAGGTTAACCAGTGCACGACCTGCCAG
129A-F	GGCAAAATCATGGCAGCACCACCAGGTATCGCAACTC
129C-F	GGCAAAATCATGGCATGCCCACCAGGTATCGCAACTC
129D-F	GGCAAAATCATGGCAGATCCACCAGGTATCGCAACTC
129E-F	GGCAAAATCATGGCAGAACCACCAGGTATCGCAACTC
129F-F	GGCAAAATCATGGCATTTCCACCAGGTATCGCAACTC
129G-F	GGCAAAATCATGGCAGGTCCACCAGGTATCGCAACTC
129H-F	GGCAAAATCATGGCACATCCACCAGGTATCGCAACTC
129K-F	GGCAAAATCATGGCAAAACCACCAGGTATCGCAACTC
129L-F	GGCAAAATCATGGCACTGCCACCAGGTATCGCAACTC
129M-F	GGCAAAATCATGGCAATGCCACCAGGTATCGCAACTC
129N-F	GGCAAAATCATGGCAAACCCACCAGGTATCGCAACTC
129P-F	GGCAAAATCATGGCACCGCCACCAGGTATCGCAACTC
129Q-F	GGCAAAATCATGGCACAGCCACCAGGTATCGCAACTC
129R-F	GGCAAAATCATGGCACGTCCACCAGGTATCGCAACTC
129S-F	GGCAAAATCATGGCAAGCCCACCAGGTATCGCAACTC
129T-F	GGCAAAATCATGGCAACTCCACCAGGTATCGCAACTC
129V-F	GGCAAAATCATGGCAGTTCCACCAGGTATCGCAACTC
129W-F	GGCAAAATCATGGCATGGCCACCAGGTATCGCAACTC
129Y-F	GGCAAAATCATGGCATATCCACCAGGTATCGCAACTC
129-R	TGCCATGATTTTGCCATCCAGGTAATCGATACGATGATCTG

180A-F	CACGATATCGCACTGGCGACTGGTATGTATGGCATGATTGC
180C-F	CACGATATCGCACTGTGCACTGGTATGTATGGCATGATTGC
180D-F	CACGATATCGCACTGGATACTGGTATGTATGGCATGATTGC
180E-F	CACGATATCGCACTGGAAACTGGTATGTATGGCATGATTGC
180F-F	CACGATATCGCACTGTTTACTGGTATGTATGGCATGATTGC
180G-F	CACGATATCGCACTGGGTACTGGTATGTATGGCATGATTGC
180H-F	CACGATATCGCACTGCATACTGGTATGTATGGCATGATTGC
180I-F	CACGATATCGCACTGATTACTGGTATGTATGGCATGATTGC
180K-F	CACGATATCGCACTGAAAACTGGTATGTATGGCATGATTGC
180M-F	CACGATATCGCACTGATGACTGGTATGTATGGCATGATTGC
180N-F	CACGATATCGCACTGAACACTGGTATGTATGGCATGATTGC
180P-F	CACGATATCGCACTGCCGACTGGTATGTATGGCATGATTGC
180Q-F	CACGATATCGCACTGCAGACTGGTATGTATGGCATGATTGC
180R-F	CACGATATCGCACTGCGTACTGGTATGTATGGCATGATTGC
180S-F	CACGATATCGCACTGAGCACTGGTATGTATGGCATGATTGC
180T-F	CACGATATCGCACTGACCACTGGTATGTATGGCATGATTGC
180V-F	CACGATATCGCACTGGTGACTGGTATGTATGGCATGATTGC
180W-F	CACGATATCGCACTGTGGACTGGTATGTATGGCATGATTGC
180Y-F	CACGATATCGCACTGTATACTGGTATGTATGGCATGATTGC
180-R	CAGTGCGATATCGTGCAGAGATGCCAGACCTGC
183A-R	CTGCAATCATGCCATACGCACCAGTCAGCAGTGCGATATC
183C-R	CTGCAATCATGCCATAGCAACCAGTCAGCAGTGCGATATC
183D-R	CTGCAATCATGCCATAATCACCAGTCAGCAGTGCGATATC
183E-R	CTGCAATCATGCCATATTCACCAGTCAGCAGTGCGATATC
183F-R	CTGCAATCATGCCATAAAAACCAGTCAGCAGTGCGATATC
183G-R	CTGCAATCATGCCATAGCCACCAGTCAGCAGTGCGATATC
183H-R	CTGCAATCATGCCATAATGACCAGTCAGCAGTGCGATATC
183I-R	CTGCAATCATGCCATAAATACCAGTCAGCAGTGCGATATC
183K-R	CTGCAATCATGCCATATTTACCAGTCAGCAGTGCGATATC
183L-R	CTGCAATCATGCCATACAGACCAGTCAGCAGTGCGATATC
183N-R	CTGCAATCATGCCATAGTTACCAGTCAGCAGTGCGATATC
183P-R	CTGCAATCATGCCATACGGACCAGTCAGCAGTGCGATATC
183Q-R	CTGCAATCATGCCATACTGACCAGTCAGCAGTGCGATATC
183R-R	CTGCAATCATGCCATAACGACCAGTCAGCAGTGCGATATC
183S-R	CTGCAATCATGCCATAGCTACCAGTCAGCAGTGCGATATC
183T-R	CTGCAATCATGCCATAGGTACCAGTCAGCAGTGCGATATC
183V-R	CTGCAATCATGCCATACACACCAGTCAGCAGTGCGATATC
183W-R	CTGCAATCATGCCATACCAACCAGTCAGCAGTGCGATATC
183Y-R	CTGCAATCATGCCATAATAACCAGTCAGCAGTGCGATATC
183-F	TATGGCATGATTGCAGGTATCCTGCAGGCATTCGC
184A-F	CTGCTGACTGGTATGGCGGGCATGATTGCAGGTATCCTG
184C-F	CTGCTGACTGGTATGTGCGGCATGATTGCAGGTATCCTG
184D-F	CTGCTGACTGGTATGGATGGCATGATTGCAGGTATCCTG
184E-F	CTGCTGACTGGTATGGAAGGCATGATTGCAGGTATCCTG

184F-F	CTGCTGACTGGTATGTTTGGCATGATTGCAGGTATCCTG
184G-F	CTGCTGACTGGTATGGGTGGCATGATTGCAGGTATCCTG
184H-F	CTGCTGACTGGTATGCATGGCATGATTGCAGGTATCCTG
184I-F	CTGCTGACTGGTATGATTGGCATGATTGCAGGTATCCTG
184K-F	CTGCTGACTGGTATGAAAGGCATGATTGCAGGTATCCTG
184L-F	CTGCTGACTGGTATGCTGGGCATGATTGCAGGTATCCTG
184M-F	CTGCTGACTGGTATGATGGGCATGATTGCAGGTATCCTG
184N-F	CTGCTGACTGGTATGAACGGCATGATTGCAGGTATCCTG
184P-F	CTGCTGACTGGTATGCCGGGCATGATTGCAGGTATCCTG
184Q-F	CTGCTGACTGGTATGCAGGGCATGATTGCAGGTATCCTG
184R-F	CTGCTGACTGGTATGCGTGGCATGATTGCAGGTATCCTG
184S-F	CTGCTGACTGGTATGAGCGGCATGATTGCAGGTATCCTG
184T-F	CTGCTGACTGGTATGACCGGCATGATTGCAGGTATCCTG
184V-F	CTGCTGACTGGTATGGTGGGCATGATTGCAGGTATCCTG
184W-F	CTGCTGACTGGTATGTGGGGCATGATTGCAGGTATCCTG
184-R	CATACCAGTCAGCAGTGCGATATCGTGCAGAGATG
214A-F	CCAATGCTGACCAACGCACTGACTGGTATGGCTCATTCTGTTG
214C-F	CCAATGCTGACCAACTGCCTGACTGGTATGGCTCATTCTGTTG
214D-F	CCAATGCTGACCAACGATCTGACTGGTATGGCTCATTCTGTTG
214E-F	CCAATGCTGACCAACGAACTGACTGGTATGGCTCATTCTGTTG
214F-F	CCAATGCTGACCAACTTTCTGACTGGTATGGCTCATTCTGTTG
214G-F	CCAATGCTGACCAACGGTCTGACTGGTATGGCTCATTCTGTTG
214H-F	CCAATGCTGACCAACCATCTGACTGGTATGGCTCATTCTGTTG
214I-F	CCAATGCTGACCAACATTCTGACTGGTATGGCTCATTCTGTTG
214K-F	CCAATGCTGACCAACAAACTGACTGGTATGGCTCATTCTGTTG
214L-F	CCAATGCTGACCAACCTGCTGACTGGTATGGCTCATTCTGTTG
214M-F	CCAATGCTGACCAACATGCTGACTGGTATGGCTCATTCTGTTG
214N-F	CCAATGCTGACCAACAACCTGACTGGTATGGCTCATTCTGTTG
214P-F	CCAATGCTGACCAACCCGCTGACTGGTATGGCTCATTCTGTTG
214Q-F	CCAATGCTGACCAACCAGCTGACTGGTATGGCTCATTCTGTTG
214R-F	CCAATGCTGACCAACCGTCTGACTGGTATGGCTCATTCTGTTG
214S-F	CCAATGCTGACCAACAGCCTGACTGGTATGGCTCATTCTGTTG
214T-F	CCAATGCTGACCAACACCCTGACTGGTATGGCTCATTCTGTTG
214V-F	CCAATGCTGACCAACGTTCTGACTGGTATGGCTCATTCTGTTG
214Y-F	CCAATGCTGACCAACTATCTGACTGGTATGGCTCATTCTGTTG
214-R	GTTGGTCAGCATTGGTGCCAGATCACCTGCTGG
240A-F	GAAACCGGTGTTGTTGCGAACCTGGCACATCAGTCTC
240C-F	GAAACCGGTGTTGTTTGCAACCTGGCACATCAGTCTC
240D-F	GAAACCGGTGTTGTTGATAACCTGGCACATCAGTCTC
240E-F	GAAACCGGTGTTGTTGAAAACCTGGCACATCAGTCTC
240F-F	GAAACCGGTGTTGTTTTCAACCTGGCACATCAGTCTC
240G-F	GAAACCGGTGTTGTTGGTAACCTGGCACATCAGTCTC
240H-F	GAAACCGGTGTTGTTCATAACCTGGCACATCAGTCTC
240I-F	GAAACCGGTGTTGTTATCAACCTGGCACATCAGTCTC

240K-F	GAAACCGGTGTTGTTAAAAACCTGGCACATCAGTCTC
240L-F	GAAACCGGTGTTGTTCTGAACCTGGCACATCAGTCTC
240M-F	GAAACCGGTGTTGTTATGAACCTGGCACATCAGTCTC
240N-F	GAAACCGGTGTTGTTAACAACCTGGCACATCAGTCTC
240P-F	GAAACCGGTGTTGTTCCGAACCTGGCACATCAGTCTC
240Q-F	GAAACCGGTGTTGTTCAGAACCTGGCACATCAGTCTC
240R-F	GAAACCGGTGTTGTTCGTAACCTGGCACATCAGTCTC
240S-F	GAAACCGGTGTTGTTAGCAACCTGGCACATCAGTCTC
240T-F	GAAACCGGTGTTGTTACCAACCTGGCACATCAGTCTC
240V-F	GAAACCGGTGTTGTTGTGAACCTGGCACATCAGTCTC
240W-F	GAAACCGGTGTTGTTTGGAACCTGGCACATCAGTCTC
240Y-F	GAAACCGGTGTTGTTTATAACCTGGCACATCAGTCTC
240-R	AACAACACCGGTTTCGTAATCACCAGTATCAATCTGCTGTG
248A-R	AACCAGTTTCGCAAACGCATGAGACTGATGTGCCAGGT
248C-R	AACCAGTTTCGCAAAGCAATGAGACTGATGTGCCAGGT
248D-R	AACCAGTTTCGCAAAATCATGAGACTGATGTGCCAGGT
248E-R	AACCAGTTTCGCAAATTCATGAGACTGATGTGCCAGGT
248F-R	AACCAGTTTCGCAAAAAAATGAGACTGATGTGCCAGGT
248H-R	AACCAGTTTCGCAAAATGATGAGACTGATGTGCCAGGT
248I-R	AACCAGTTTCGCAAAAATATGAGACTGATGTGCCAGGT
248K-R	AACCAGTTTCGCAAATTTATGAGACTGATGTGCCAGGT
248L-R	AACCAGTTTCGCAAACAGATGAGACTGATGTGCCAGGT
248M-R	AACCAGTTTCGCAAACATATGAGACTGATGTGCCAGGT
248N-R	AACCAGTTTCGCAAAGTTATGAGACTGATGTGCCAGGT
248P-R	AACCAGTTTCGCAAACGGATGAGACTGATGTGCCAGGT
248Q-R	AACCAGTTTCGCAAACTGATGAGACTGATGTGCCAGGT
248R-R	AACCAGTTTCGCAAAACGATGAGACTGATGTGCCAGGT
248S-R	AACCAGTTTCGCAAAGCTATGAGACTGATGTGCCAGGT
248T-R	AACCAGTTTCGCAAAGGTATGAGACTGATGTGCCAGGT
248V-R	AACCAGTTTCGCAAACACATGAGACTGATGTGCCAGGT
248W-R	AACCAGTTTCGCAAACCAATGAGACTGATGTGCCAGGT
248Y-R	AACCAGTTTCGCAAAATAATGAGACTGATGTGCCAGGT
248-F	TTTGCGAAACTGGTTCAGGCTGGTGAAGATCAGG
249A-F	ACATCAGTCTCATGGTGCGGCGAAACTGGTTCAGGCT
249C-F	ACATCAGTCTCATGGTTGCGCGAAACTGGTTCAGGCT
249D-F	ACATCAGTCTCATGGTGATGCGAAACTGGTTCAGGCT
249E-F	ACATCAGTCTCATGGTGAAGCGAAACTGGTTCAGGCT
249G-F	ACATCAGTCTCATGGTGGTGCGAAACTGGTTCAGGCT
249H-F	ACATCAGTCTCATGGTCATGCGAAACTGGTTCAGGCT
249I-F	ACATCAGTCTCATGGTATTGCGAAACTGGTTCAGGCT
249K-F	ACATCAGTCTCATGGTAAAGCGAAACTGGTTCAGGCT
249L-F	ACATCAGTCTCATGGTCTGGCGAAACTGGTTCAGGCT
249M-F	ACATCAGTCTCATGGTATGGCGAAACTGGTTCAGGCT
249N-F	ACATCAGTCTCATGGTAACGCGAAACTGGTTCAGGCT

249P-F	ACATCAGTCTCATGGTCCGGCGAAACTGGTTCAGGCT
249Q-F	ACATCAGTCTCATGGTCAGGCGAAACTGGTTCAGGCT
249R-F	ACATCAGTCTCATGGTCGTGCGAAACTGGTTCAGGCT
249S-F	ACATCAGTCTCATGGTAGCGCGAAACTGGTTCAGGCT
249T-F	ACATCAGTCTCATGGTACCGCGAAACTGGTTCAGGCT
249V-F	ACATCAGTCTCATGGTGTTGCGAAACTGGTTCAGGCT
249W-F	ACATCAGTCTCATGGTTGGGCGAAACTGGTTCAGGCT
249Y-F	ACATCAGTCTCATGGTTATGCGAAACTGGTTCAGGCT
249-R	ACCATGAGACTGATGTGCCAGGTTGAAAACAACACC

Sequence IDs of the Panel of 134 IREDs

Table S11. The sequence IDs of the panel of 134 IREDs.

No.	Sequence ID	No.	Sequence ID
IRED-2	WP_225795959.1	IRED-114	MCC5941871.1
IRED-3	WP_056651502.1	IRED-115	XP_033408764.1
IRED-5	WP_225993023.1	IRED-116	KAE8374192.1
IRED-6	WP_184074226.1	IRED-117	PZN88780.1
IRED-8	WP_179839446.1	IRED-118	WP_127591777.1
IRED-12	WP_249674623.1	IRED-119	WP_075124000.1
IRED-13	WP_181132505.1	IRED-120	MBD0745557.1
IRED-17	WP_233599896.1	IRED-121	WP_128507713.1
IRED-18	WP_068897902.1	IRED-124	WP_026123258.1
IRED-19	WP_121434681.1	IRED-125	WP_221096248.1
IRED-20	WP_242905576.1	IRED-126	WP_221155618.1
IRED-22	WP_179646347.1	IRED-127	WP_129648975.1
IRED-23	RWK41252.1	IRED-129	WP_014765313.1
IRED-24	WP_131903132.1	IRED-130	WP_081158374.1
IRED-25	WP_163855277.1	IRED-131	WP_100671813.1
IRED-27	WP_203858680.1	IRED-132	UIJ94070.1
IRED-28	WP_254043653.1	IRED-133	WP_065786391.1
IRED-31	WP_153418794.1	IRED-134	WP_209664744.1
IRED-32	WP_184698759.1	IRED-135	MBN9384696.1
IRED-33	WP_078439580.1	IRED-136	WP_184872353.1
IRED-34	WP_215078439.1	IRED-138	WP_131465700.1
IRED-36	WP_189833181.1	IRED-139	WP_141854780.1
IRED-37	WP_030547959.1	IRED-140	WP_132318745.1
IRED-42	WP_078439580.1	IRED-142	WP_184732442.1
IRED-44	WP_105670645.1	IRED-143	WP_253670862.1
IRED-45	WP_084749945.1	IRED-144	WP_093683835.1
IRED-47	XP_031921959.1	IRED-145	QKW13528.1
IRED-48	KAE8418542.1	IRED-146	WP_189951058.1
IRED-49	KAF4226275.1	IRED-147	WP_188302856.1
IRED-50	WP_242905576.1	IRED-148	WP_027941321.1
IRED-51	WP_119730833.1	IRED-149	WP_110628025.1
IRED-52	WP_099877301.1	IRED-150	WP_030900598.1
IRED-54	WP_200394112.1	IRED-151	WP_114530622.1
IRED-55	GHH51860.1	IRED-152	WP_228452861.1
IRED-56	WP_121006980.1	IRED-153	WP_132118894.1
IRED-59	WP_120005127.1	IRED-154	OMI34305.1
IRED-60	WP_104980346.1	IRED-155	WP_060948637.1
IRED-61	WP_253870844.1	IRED-156	WP_246025534.1
IRED-64	WP_033246350.1	IRED-157	WP_132480472.1

IRED-66	WP_195075817.1	IRED-158	WP_093155550.1
IRED-67	WP_147256524.1	IRED-159	WP_029114970.1
IRED-68	WP_046770251.1	IRED-160	WP_076169018.1
IRED-69	WP_184074226.1	IRED-161	WP_072027083.1
IRED-70	WP_243390846.1	IRED-162	WP_192744171.1
IRED-72	WP_151479989.1	IRED-163	WP_125795693.1
IRED-73	RLU86845.1	IRED-164	WP_023809753.1[2]
IRED-74	WP_247765940.1	IRED-165	WP_205760981.1[3]
IRED-76	WP_053733177.1	IRED-168	WP_179754454.1
IRED-81	MBN6053481.1	IRED-169	WP_121222095.1
IRED-83	WP_101787845.1	IRED-170	WP_185129816.1
IRED-84	MCS3801987.1	IRED-171	WP_158581372.1
IRED-93	WP_151484701.1	IRED-172	WP_189224997.1
IRED-97	WP_237773465.1	IRED-174	WP_192702180.1
IRED-98	KAF4266800.1	IRED-176	WP_152775210.1
IRED-99	KEY77803.1	IRED-179	XP_001827659.1[4]
IRED-100	XP_001266407.1	IRED-180	WP_019888617.1[5]
IRED-101	XP_043147525.1	IRED-181	WP_028934187.1
IRED-102	KAF4211801.1	IRED-182	UPK83284.1[6]
IRED-104	XP_001276415.1	IRED-184	WP_069164777.1
IRED-105	UIS96764.1	IRED-185	AKF85151.1[7]
IRED-107	KAB8068570.1	IRED-186	7OG3_A[8]
IRED-108	KAG2414157.1	IRED-188	WP_088993565.1[9]
IRED-109	KAF4226275.1	IRED-189	WP_021595532.1[10]
IRED-110	XP_002626263.1	IRED-191	WP_007128231.1[11]
IRED-111	KAF9279940.1	IRED-192	4OQY_A[12]
IRED-112	KAI8363422.1	IRED-194	WP_185129816.1
IRED-113	KAF9159830.1	IRED-195	7WNW_A[13]

References

[1] Gu, G.; Xu, Z.; Wen, L.; Liang, J.; Wang, C.; Wan, X.; Zhao, Y. Chirality Sensing of N-Heterocycles via ^{19}F NMR. *JACS Au* **2023**, *3*, 1348-1357.

[2] France, S. P.; Howard, R. M.; Steflik, J.; Weise, N. J.; Mangas-Sanchez, J.; Montgomery, S. L.; Crook, R.; Kumar, R.; Turner, N. J. Identification of Novel Bacterial Members of the Imine Reductase Enzyme Family that Perform Reductive Amination. *ChemCatChem.* **2018**, *10*, 510-514.

[3] Zhan, Z. Z.; Xu, Z. F.; Yu, S. S.; Feng, J. H.; Liu, F. F.; Yao, P. Y.; Wu, Q. Q.; Zhu, D. M. Stereocomplementary Synthesis of a Key Intermediate for Tofacitinib via Enzymatic Dynamic Kinetic Resolution-Reductive Amination. *Adv. Synth. Catal.* **2022**, *364*, 2380– 2386

[4] Aleku, G. A.; France, S. P.; Man, H.; Mangas-Sanchez, J.; Montgomery, S. L.; Sharma, M.; Leipold, F.; Hussain, S.; Grogan, G.; Turner, N. J. A reductive aminase from Aspergillus oryzae. *Nat. Chem.* **2017**, *9*, 961−969.

[5] Kumar, R.; Karmilowicz, M. J.; Burke, D.; Burns, M.; Clark, L. A.; Connor, C. G.; Cordi, E.; Do, N. M.; Doyle, K. M.; Hoagland, S.; Lewis, C. A.; Mangan, D.; Martinez, C. A.; McInturff, E. L.; Meldrum, K.; Pearson, R.; Steflik, J.; Rane, A.; Weaver, J. Biocatalytic reductive amination from discovery to commercial manufacturing applied to abrocitinib JAK1 inhibitor. *Nat. Catal.* **2021**, *4*, 775−782.

[6] Thorpe, T. W.; Marshall, J. R.; Harawa, V.; Ruscoe, R. E.; Cuetos, A.; Finnigan, J. D.; Angelastro, A.; Heath, R. S.; Parmeggiani, F.; Charnock, S. J.; Howard, R. M.; Kumar, R.; Daniels, D. S. B.; Grogan, G.; Turner, N. J. Multifunctional biocatalyst for conjugate reduction and reductive amination. *Nature* **2022**, *604*, 86−91.

[7] Roiban, G. D.; Kern, M.; Brown, M. J. B., Efficient Biocatalytic Reductive Aminations by Extending the Imine Reductase Toolbox. *ChemCatChem.* **2017**, *9*, 4475−4479.

[8] Ma, E. J.; Siirola, E.; Moore, C.; Kummer, A.; Stoeckli, M.; Faller, M.; Bouquet, C.; Eggimann, F.; Ligibel, M.; Huynh, D.; Cutler, G.; Siegrist, L.; Lewis, R. A.; Acker, A. C.; Freund, E.; Koch, E.; Vogel, M.; Schlingensiepen, H.; Oakeley, E. J.; Snajdrova, R. Machine-Directed Evolution of an Imine Reductase for Activity and Stereoselectivity. *ACS Catal.* **2021**, *11*, 12433−12445.

[9] Xu, Z.; Yao, P.; Sheng, X.; Li, J.; Li, J.; Yu, S.; Feng, J.; Wu, Q.; Zhu, D. Biocatalytic Access to 1,4-Diazepanes via Imine Reductase-Catalyzed Intramolecular Asymmetric Reductive Amination. *ACS Catal.* **2020**, *10*, 8780−8787.

[10] Zhu, J. M.; Tan, H. Q.; Yang, L.; Dai, Z.; Zhu, L.; Ma, H. M.; Deng, Z. X.; Tian, Z. H.; Qu, X. D. Enantioselective Synthesis of 1-Aryl-Substituted Tetrahydroisoquinolines Employing Imine Reductase. *ACS Catal.* **2017**, *7*, 7003−7007.

[11] Li, H.; Luan, Z. J.; Zheng, G. W.; Xu, J. H. Efficient Synthesis of Chiral Indolines using an Imine Reductase from Paenibacillus lactis. *Adv. Synth. Catal.* **2015**, *357*, 1692−1696.

[12] Leipold, F.; Hussain, S.; Ghislieri, D.; Turner, N. J. Asymmetric Reduction of Cyclic Imines Catalyzed by a Whole-Cell Biocatalyst Containing an (S)-Imine Reductase. *ChemCatChem.* **2013**, *5*, 3505−3508.

[13] Zhang, J.; Liao, D. H.; Chen, R. C.; Zhu, F. F.; Ma, Y. Q.; Gao, L.; Qu, G.; Cui, C. S.; Sun, Z. T.; Lei, X. G.; Gao, S. S. Tuning an Imine Reductase for the Asymmetric Synthesis of Azacycloalkylamines by Concise Structure-Guided Engineering. *Angew. Chem., Int. Ed.* **2022**, *61*, e202201908.

^{1}H and ^{19}F NMR Spectra of probe-CF_3

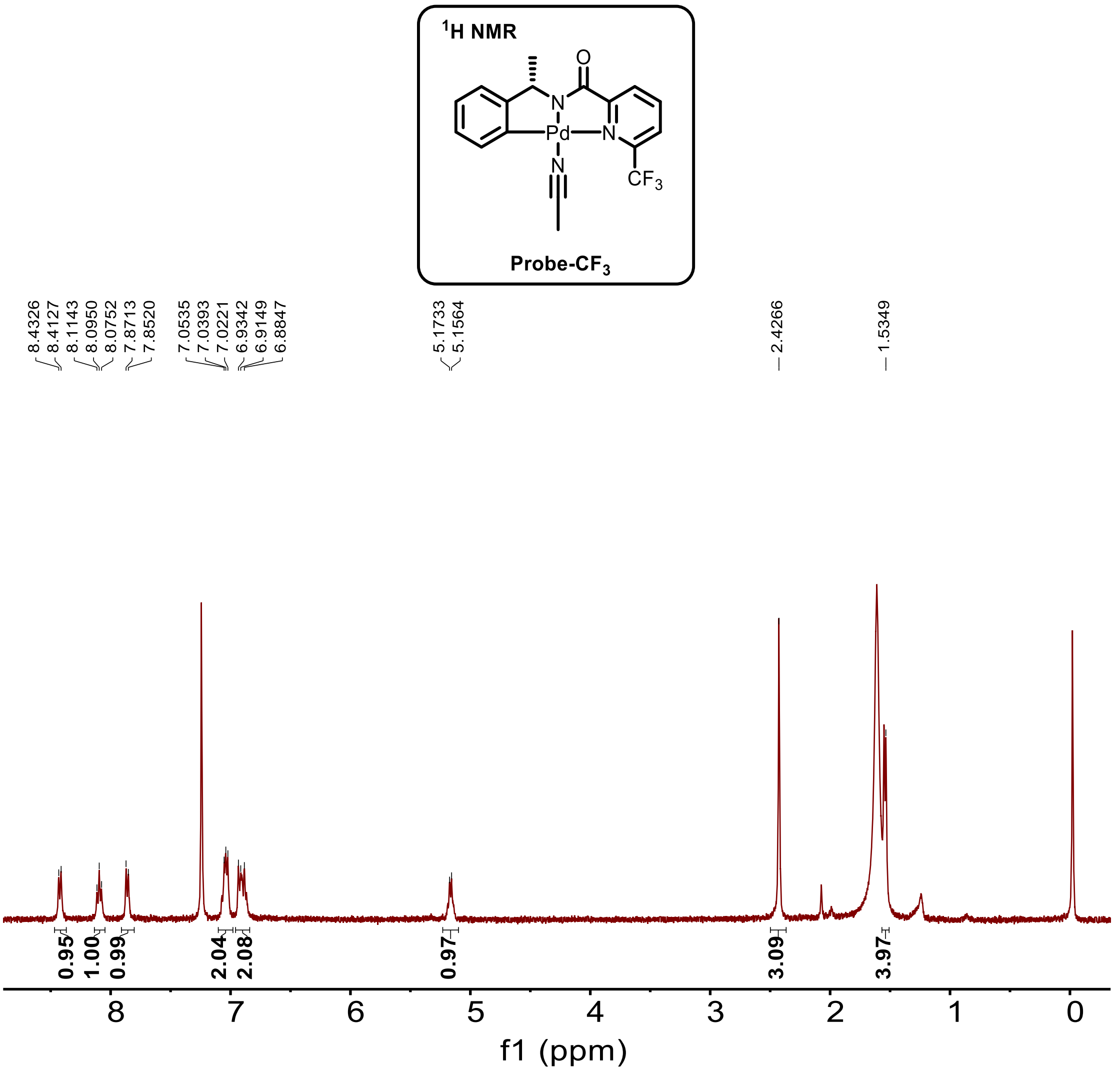

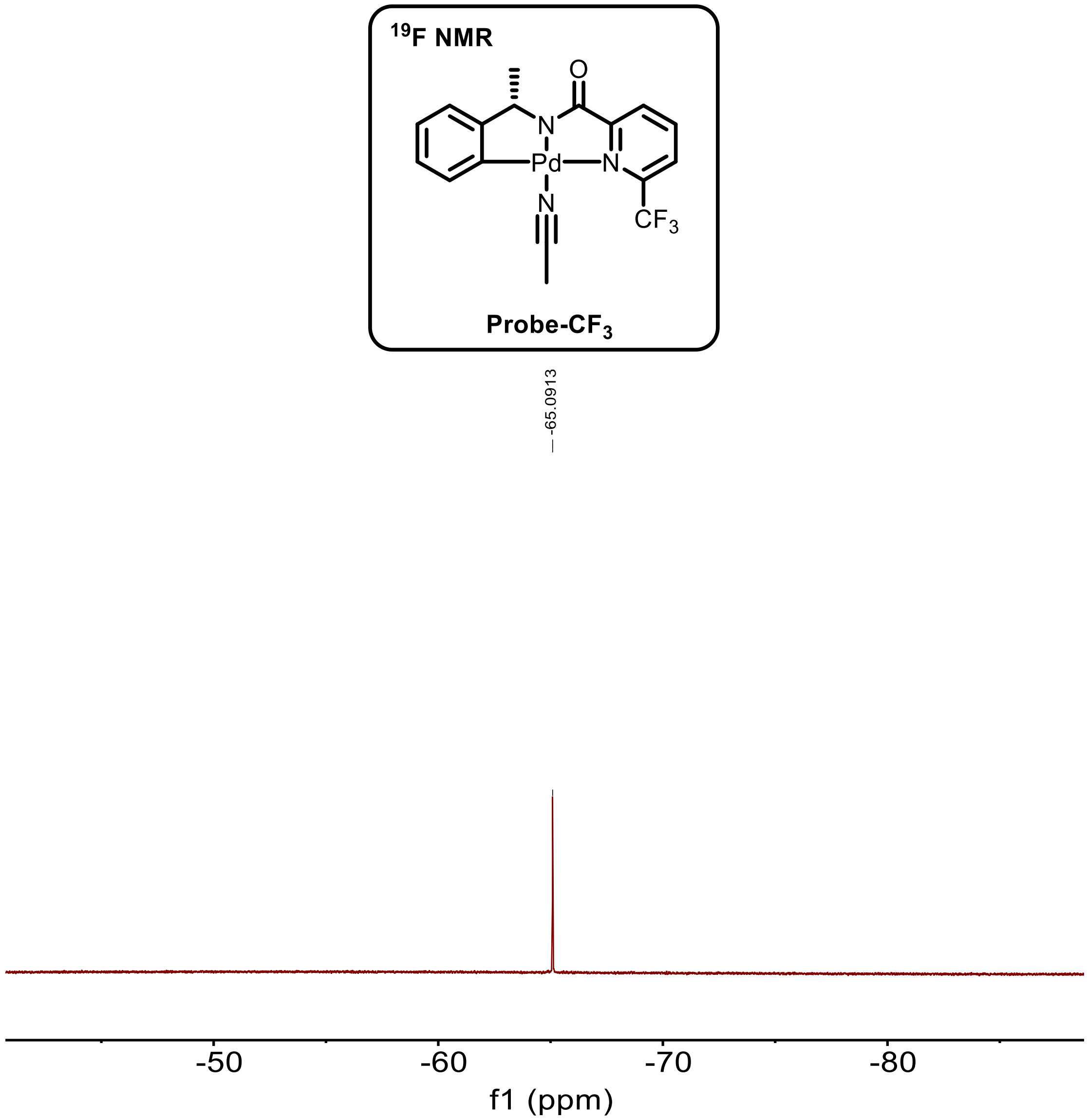

19F NMR
Probe-CF3
-65.0913
-50
-60
-70
-80
f1 (ppm)
